# Test Methods for Measuring the Electrical Output of Electroshock Weapons

**Published:** 2016-07

**Authors:** Nicholas G Paulter, David Jenkins, Norimitsu Ichikawa, Michael Leonesio

**Affiliations:** 1 National Institute of Standards and Technology, 100 Bureau Drive, Gaithersburg, MD 20899, USA; 2 The Pennsylvania State University, P. O. Box 30, State College, PA 16804-0030, USA; 3 Kogakuin University, 1-24-2 Nishishinjuku, Shinjuku, Tokyo 163-8677, Japan; 4 Leonesio Consulting, LLC, 412 S. White St., Suite 210, Athens, TN 37303, Greece

**Keywords:** Conducted energy weapon, Electroshock weapon, IEC, Incapacitation, Neuromuscular disruption, Standard test method

## Abstract

Electroshock weapons (ESWs) have become a ubiquitous component of weapons used in the arsenal of domestic law enforcement (LE) agencies and the military around the world. It is a major contributor to the escalation of force policies and procedures of many LE agencies. ESWs function by providing a high-voltage low-current electrical shock that, when discharged into a live (typically human) target, can temporarily incapacitate that target. Consequently, it is important to accurately measure the output of the ESW to ensure it is operating properly. Moreover, accurate and standardized measurements of ESW output support collaborative transfer of information, thus facilitating the advancement of the knowledge of physiological ESW effects, promoting the advancement of the technology for safe and effective use, and facilitating accurate comparison of ESW performance.

## Introduction

The electroshock weapon (ESW), also known as a neuromuscular incapacitator, conducted energy weapon, conducted energy device, etc., is one component of a continuum of force that can be applied by law enforcement and military to control or subdue an assailant or combative person. In typical usage, the person being subdued is not permanently harmed by the output of an ESW. Additionally, there is the expectation by the operator of the ESW that the ESW is capable of incapacitating a targeted person. To ensure that safe usage and incapacitation effectiveness are achieved requires knowledge of the physiological responses of a human to exposure from the output of an ESW, methods of accurately and reproducibly measuring the ESW output, and policies and procedures that ensure informed and skillful application of the ESW.

This article discusses the results of the research conducted to develop methods for accurately measuring ESW output. This research subsequently led to the development of the international test method standard, the “IEC 62792 Edition 1.0 2015–02, Measurement method for the output of electroshock weapons” [1]. This discussion will include describing the IEC 62792, parameters describing ESW electrical outputs, methods for measuring these parameters, and how the computation of these parameters can affect measurement results. This article will not consider physiological responses of humans to ESW exposure or policies and procedures for ESW employment.

It is essential that the parameters used to characterize the electrical output of the ESW are accurately described, as this maximizes the reproducibility of measurements between different entities, promotes accurate communication of measurement results, promotes ESW technical advancement, and supports the assessment of its physiological effects. Consequently, the parameters used to describe ESW output are discussed, including how the values associated with these parameters are computed as this may affect comparative measurements of models or inter-laboratory comparisons and will affect the design of test methods and the selection of test instrumentation.

The test methods used to extract the waveforms are the crux of ESW metrology. However, not all test methods need to be metrological quality if the purpose is to ensure compliance of a unit to manufacturer specifications or to ensure a unit is nominally operating properly. Consequently, different levels of test methods are briefly described, each providing different levels of measurement accuracy. However, the metrological measurement system should be capable of acquiring as much of the signal content of the ESW output as possible and with the highest fidelity as possible because the physiological significance of this signal content is not completely understood.

The IEC 62792 describes methods for measuring the output of ESW, and includes terms and parameters with definitions for describing ESW electrical output and algorithms for computing parameter values. It was published in 2015. The purpose of IEC 62792, its contents, and its development are described in International test method standard of this document.

### ESW function and use

The ESW functions by delivering a high-voltage, low-current, electrical shock to an individual. This shock is typically of sufficient energy to cause the individual to become temporarily incapacitated even for a few seconds after the current discharge has been completed. As stated earlier, the ESW is one component in the continuum of force options employed by law enforcement, both civilian and military, to control a combative person or an assailant. Many countries use ESW, and the range of national-use policies is variable. Some countries outlaw their use altogether, others allow only law enforcement agencies to use them, and others also allow the citizenry to use them for self-defense. Within a country, different legal jurisdictions may have different policies regarding use of ESW. The interested reader can search the internet to learn of different national policies.

Since the deployment and employment of ESW, there have been claims of adverse effects, ranging from inadequate output for incapacitation to death. It is not the purpose of this manuscript to list or describe these claims, which can readily found on the internet, as this may lend credence to these claims. Furthermore, various conflicting discussions on the efficacy and safe use of ESW have been published, which can be found on the internet, but the veracity of these have not been confirmed. The plethora of these claims and reports demonstrates the need to establish performance limits for ESW and to establish accurate and reproducible methods for measuring the output of the ESW. The establishment of performance limits is outside the purview of this article. This article describes test methods that provide accurate and reproducible measurement of the current and high voltage outputs of ESW. Such a test method provides an objective means of communication of ESW output among ESW manufacturers, users (agencies), the medical community, and the general public.

## International Test Method Standard

The concept for an ESW test method standard was borne from a workshop on ESW that was convened in January 2011 [2]. Attendees of this workshop identified key concerns for the advancement of ESW technology and its safe and effective implementation. These concerns included knowledge of its effect (both current models and future models) on human physiology, policy on ESW deployment and use, training, and test and measurement. Since both training and policy are dependent on the knowledge of the physiological response, and the fact that the language of ESW communication and reproducibility of measurement were not sufficiently adequate to enhance this knowledge, a test method standard was identified as the first step to address the concerns articulated at the workshop.

**Figure 1:**
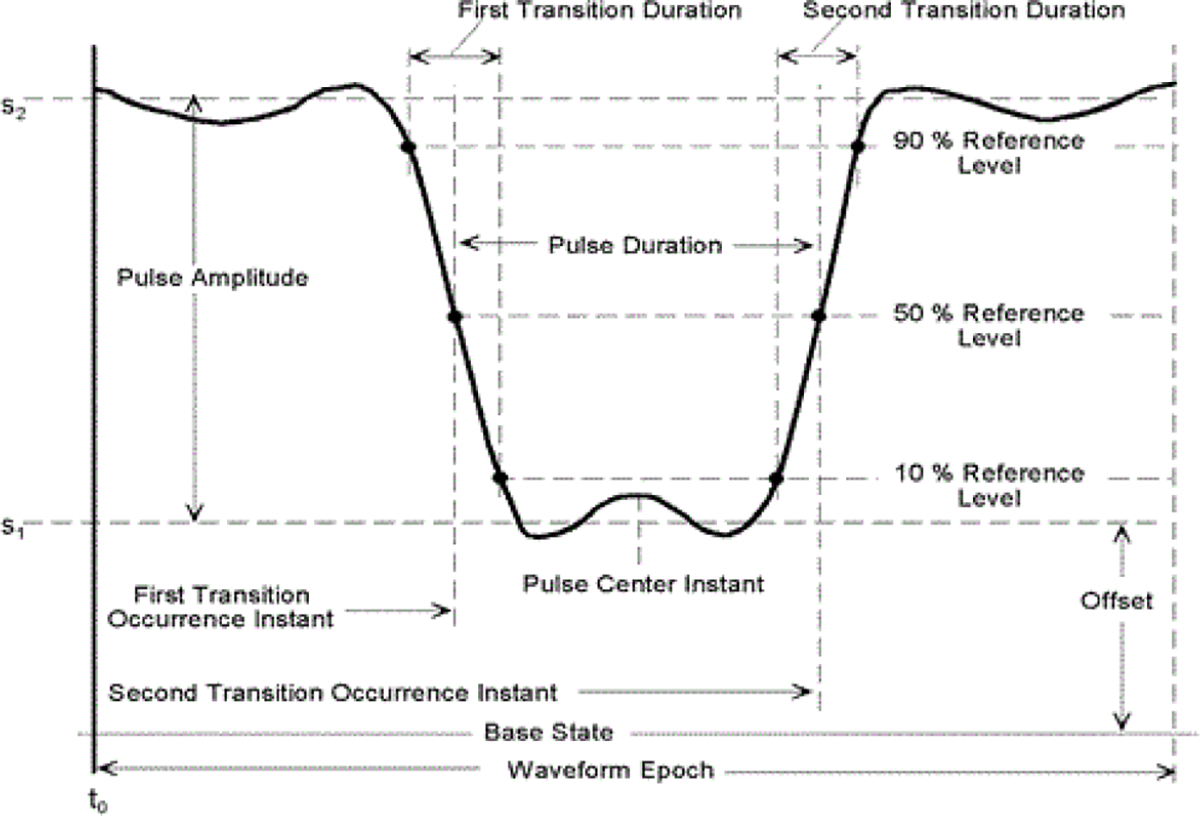
Parameters for a negative pulse waveform.

**Figure 2:**
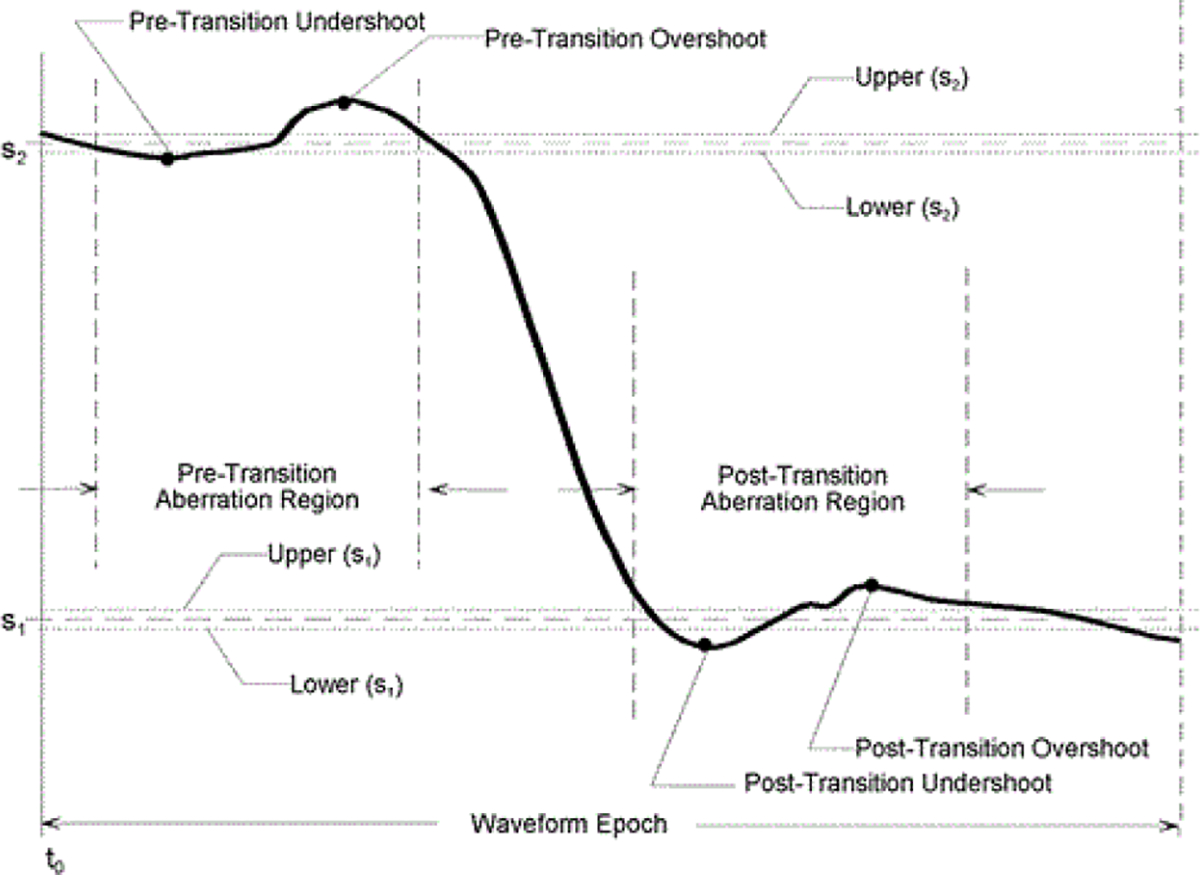
State level and aberration parameters for a negative-going single-transition wave form.

**Figure 3:**
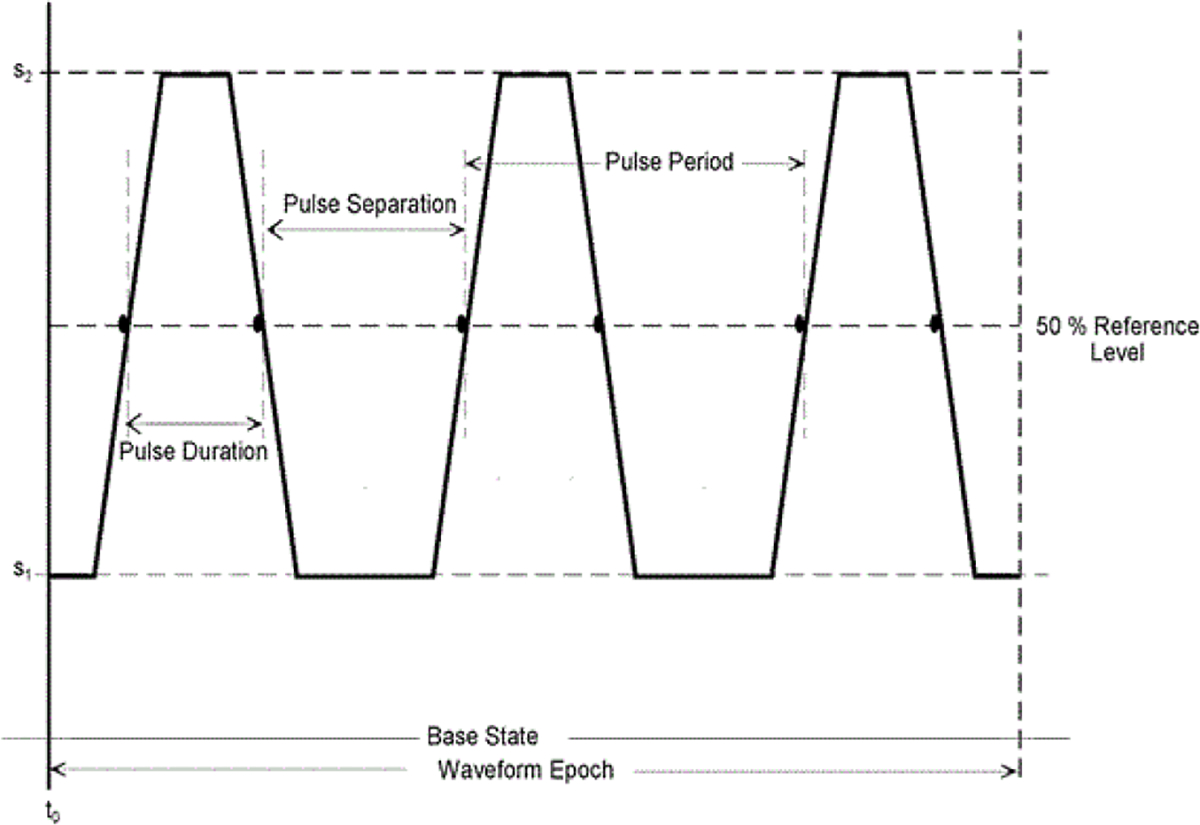
Pulse parameters for a pulse train.

Other than for the IEC 62792, there are no international test method standards for ESWs. The Canadian government produced a test method standard [3] that was written to describe methods for measuring the output for a specific manufacturer’s products at the time of its writing. In addition, there are prescribed test methods and terminology related to measurement of the ESW current and high-voltage outputs that are contained in some manufacturer specifications documents and government agency procurement requirements. The IEC 62792 provides a list of terms (parameters), their definitions, and algorithms for their computation. The terms are based on pulse parameters given in the IEC 60469, Ed. 1, 2013–04, “Transitions, pulses and related waveforms – Terms, definitions and algorithms” [4] or equivalently, the IEEE Std. 181–2011, “IEEE Standard for Transitions, Pulses, and Related Waveforms” [5]. The IEC 60469 and IEEE 181 are nominally identical and provide detailed description of pulse parameters. For example, Figure 1 demonstrates the details for some of the parameters used to describe a negative pulse waveform, and Figure 2 shows the detail on the state level and aberration nomenclature for a negative-going single transition waveform. Figure 3 shows the parameters associated with a pulse train. All the parameters shown in these figures are exhibited by the ESW high voltage and current outputs that have been examined thus far.

The IEC 62792 contains definitions of and computation algorithm for these parameters, consistent with the IEEE and IEC standards. Furthermore, the IEC 62792 contains waveform parameters unique for ESW measurement applications, where these parameters are derived from those contained in the IEC 60469 (IEEE 181). These unique parameters will be described in Effects on measurement results of this document, including the effects of user defined reference values for state levels and instants on the computation of their values. User defined reference values are allowed if the user describes, with sufficient detail to replicate the user’s results, how the reference values are different from the standard’s default reference values.

The IEC 62792 describes different measurement system configurations based on different user-required levels of measurement rigor, with nominal system performance requirements for each, and methods of calibration for these systems, all of which are described in Test methods of this document.

## Parameters to be measured

**Figure 4:**
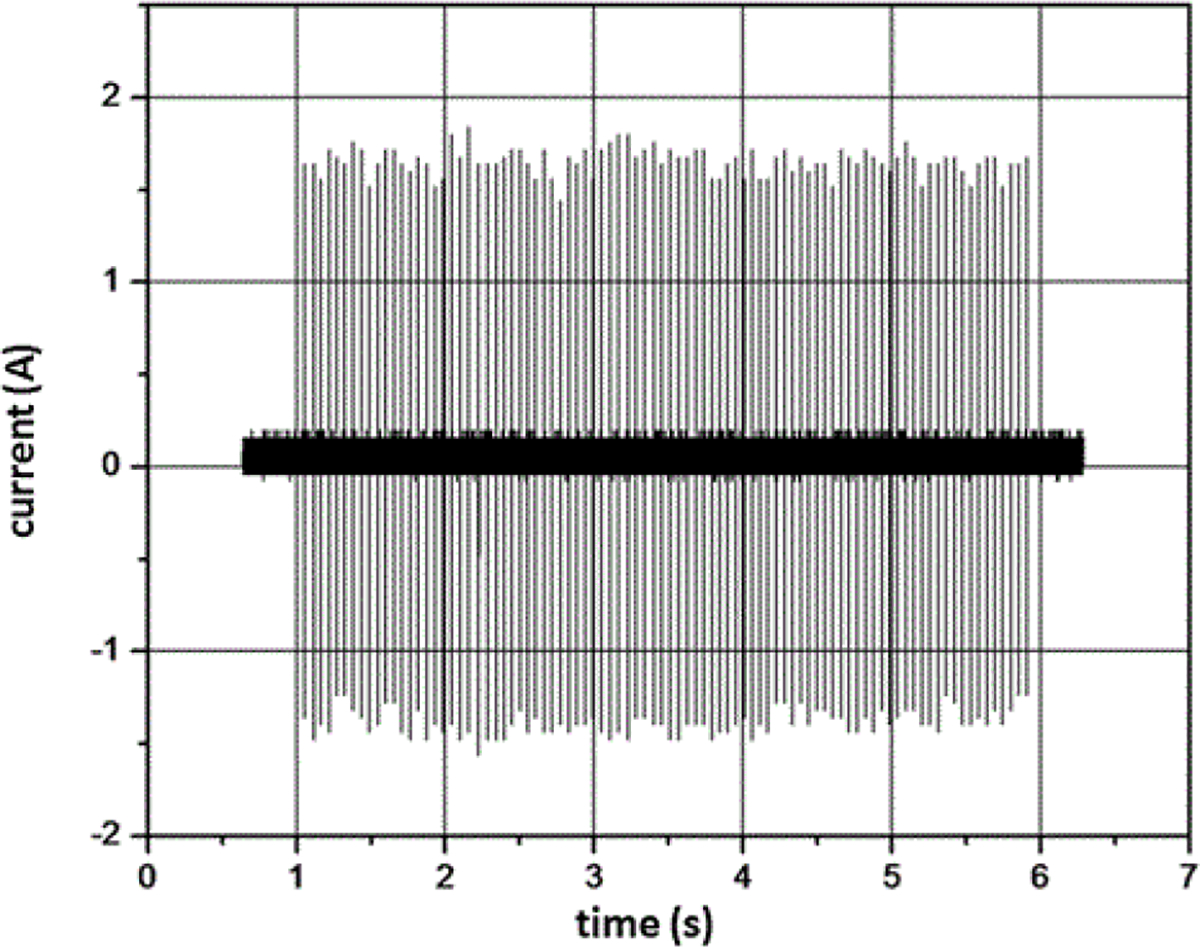
Pulse train from current output for a given model of ESW. The temporal resolution (or sampling interval) for the waveform is 1.28 μs.

**Figure 5:**
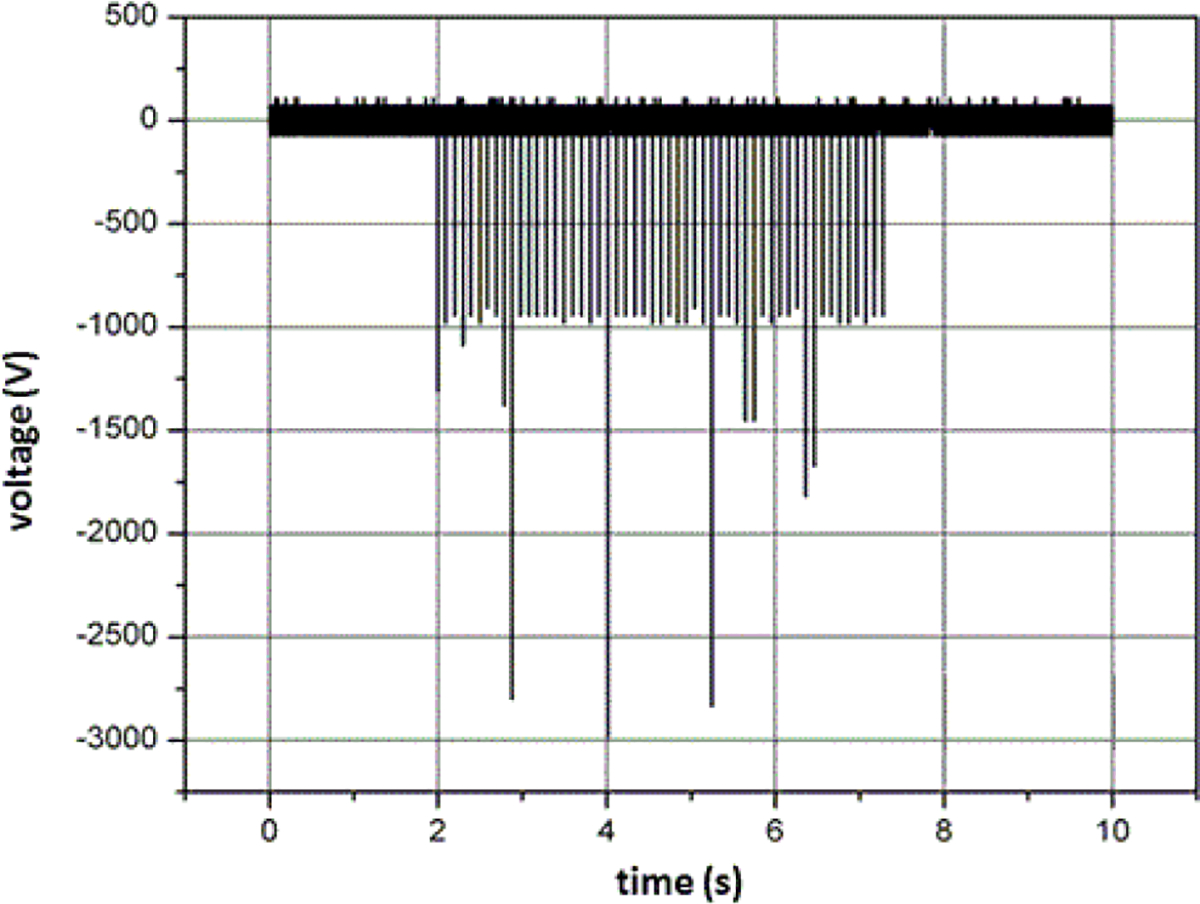
Pulse train from current output for a model of ESW different from that shown in (Figure 4). The temporal resolution (or sampling interval) for the waveform is 2.5 μs. The variation of the peak amplitude is caused by an inadequate sampling interval (or resolution).

As shown in Figures 4 and 5, the ESW output is a pulse burst containing a train of pulses. There are tens to hundreds of these nominally identical electrical pulses in the burst. This burst may last from 1 s to 2 s to more than 10 s and is not repeated unless the ESW trigger is activated. The pulses in a burst may have transition durations ranging from approximately a couple of nanoseconds to more than a few microseconds. The pulses may be bipolar and may exhibit aberrations that are electrical-load dependent. Consequently, parameters should be extracted from the pulse train that accurately and reproducibly describes the output of the ESW. The language describing these parameters and the algorithms that are used to compute the values of these parameters should be clearly understood and not be subject to misinterpretation.

The waveform parameters that are most commonly cited to describe ESW performance are peak amplitudes of voltage and/or current, total charge (net, positive, or negative), partial charge (charge contained within defined parts of the waveform), pulse duration, pulse repetition rate, number of pulses, and pulse burst duration. All of these parameters are affected by noise, sampling rate, and the attenuation bandwidth of the measurement system. Examples of these effects are discussed in Effects on measurement results of this document. The temporal parameters (durations and rates) and charge parameters will also be dependent on user-defined values for amplitude thresholds and initial and final summation instants (discussed in Effects on measurement results of this document). The following are two examples of the computation of ESW pulse parameters, pulse duration and net charge.

Pulse duration, TP, is computed using:

(1) $$T_{P}=\left|t_{2,x\text{\%}}-t_{1,x\text{\%}}\right|$$

where *t*_2,*x*%_ is the reference level instant for the first transition of the pulse, *t*_1,*x*%_ is the reference level instant for the second transition of the pulse [4,5], and where *x*% is the percent reference level specified by the user. The value of TP will be affected by changes in *t*_2,*x*%_ and *t*_1,*x*%_ from their nominal values. Similarly pulse separation, pulse period, etc., will be affected by deviation of *t*_2,*x*%_ and *t*_1,*x*%_ from their nominal values.

The charge delivered by the ESW is computed using an average value, either over the entire current waveform (for net charge) or user-specified parts of the current waveform (for positive or negative charge). The average value for a pulse in a pulse burst is computed using:

(2) $$\bar{y_{i}}=\left(\frac{1}{M_{i}}\right)\sum_{j=1}^{M_{i}}y_{i,j},j=1,\ldots ,M_{i}$$

where the *y*_*i,j*_ are the waveform values, *i* is the index for the waveform sub-epoch (one sub-epoch for each pulse in a pulse burst), *M*_*i*_ is the number of waveform samples in the *i*th waveform sub-epochs, and *j* is the sample index [1]. A waveform sub-epoch correlates to one pulse in the pulse train. For the examples that will be used here, there will be only one waveform sub-epoch used, so equation 2 reduces to:

(3) $$\bar{y}=\left(\frac{1}{M}\right)\sum_{j=1}^{M}y_{j}$$

Net charge, *Q*_*net*_, is then computed from equation 3 using:

(4) $$Q_{net}=\bar{y}T$$

where *T* is the duration of the waveform epoch or the summation interval defined by the user [1]. Measurement and computation processes and variables that affect *y*_*i*_ and *T* will alter the value of $\bar{y}$ and $Q_{net}$. Therefore, user selection of the epoch will affect *Q*_*net*_. Furthermore, common industry practice is to set amplitude thresholds and then to excluded from the summation any waveform values (the *y*_*j*_ in equation 3) that are less than (or greater than) this threshold., thereby affecting the value of *Q*_*net*_.

As mentioned in International test method standard of this document, there are currently no applicable safety standards for ESW devices. However, safety-relevant parameters can be found in existing IEC electrical safety standards [6–8]. These standards were derived from electrical accident analysis and animal testing under situations similar to that experienced by humans during exposure to electricity. Two examples are typical alternating current line voltage and capacitor discharges from electrical equipment. Both of these situations could be encountered by a technician fixing a piece of electrical equipment still connected to AC mains or with residual energy stored in a capacitor. The current and duration (charge) are shown in these IEC standards with relevancy to the electrical safety of these two situations.

## Measurement Systems and their Design

**Figure 6:**
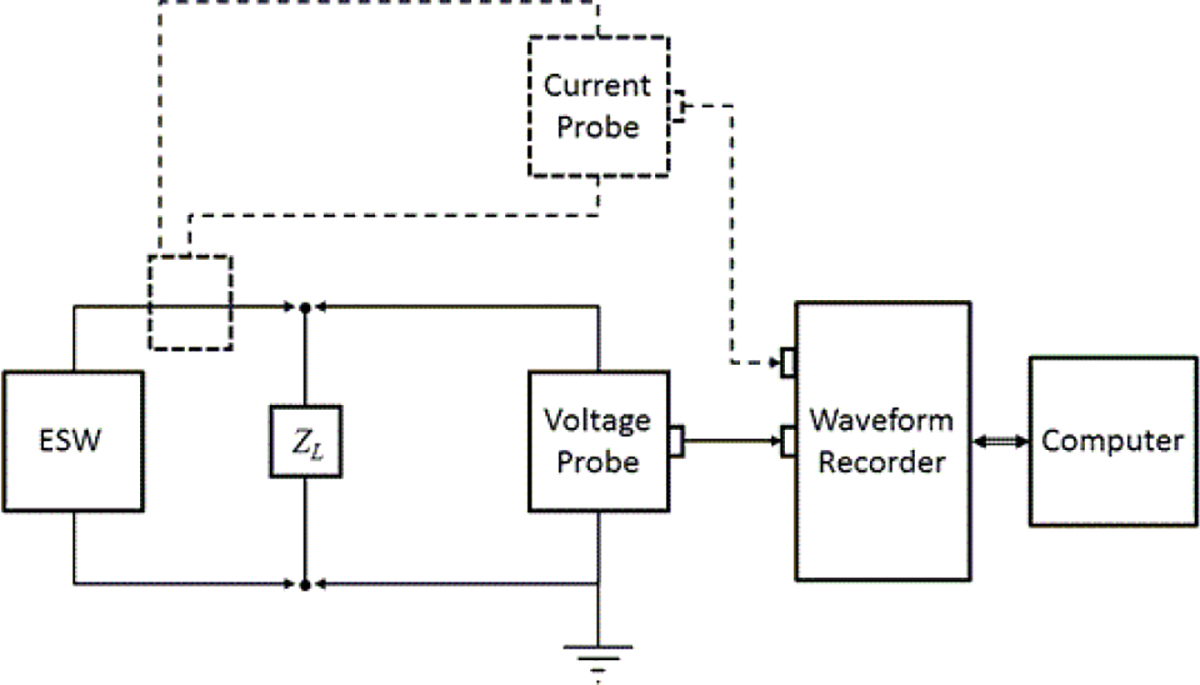
ESW measurement system.

A general schematic for an ESW measurement system is shown in Figure 6. The figure shows the ESW being tested, the electrical load into which the ESW discharges its energy, a voltage probe, and a waveform recorder. The dotted lines in Figure 6 show a measurement configuration for the measurement of the current output of the ESW. If the waveform recorder has two or more input channels, the high-voltage and current waveforms can be acquired simultaneously. If this is not the case, the high-voltage and current waveforms have to be measured sequentially. In this case, additional measurement uncertainties will occur because different pulse bursts, from independent trigger activations, will not produce identical waveforms.

A metrological test method should support acquisition of the entire ESW output with the highest fidelity possible to ensure all frequency components of the signal are collected. It is important that the metrological measurement system and process do not restrict the information collected because it is currently not well understood what information is important to and relevant for understanding the physiological effects of ESW exposure. Moreover, newer ESW designs may generate signals with greater bandwidth than do current commercially-available models. However, a metrological level measurement system is not necessarily required for all measurement applications.

### Different tiers of measurement system performance

As mentioned previously, the IEC standard allows for measurement systems offering different levels of metrological rigor, specifically, three different levels of measurement systems are allowed, as shown in Table 1. The tertiary system allows the user to define performance specifications for certain parameters, where these user-defined specifications are based on the user’s documented interpretation and knowledge of the operation of ESW and of the physiological response to the ESW’s output. The tertiary system, for example, could be used for field operational verification. In addition to the waveform recorder specifications, the cables, connectors, resistive loads, and probes also have specific requirements, as shown in Table 2.

The greatest differentiator in the performance of these measurement systems is from the waveform recorder, as most of the other components are passive. The reference system requires the ability to acquire waveforms with a nominal sample rate in excess of 1 GS/s for up to 10 s. This requires 10 GB of memory that can store data at high rates. In addition to this memory requirement, the waveform recorder should support nominally 1 GHz analog bandwidth measurements. At the time of the writing of this manuscript, these bandwidth and memory requirements are not found in a commercially-available instrument and must be obtained by a custom built waveform recorder. The amplitude and noise characteristics are not as stringent as the temporal (discussed in Temporal characteristics of measurement system of this document) and memory requirements and could be met by many instruments.

### Temporal characteristics of measurement system

**Figure 7:**
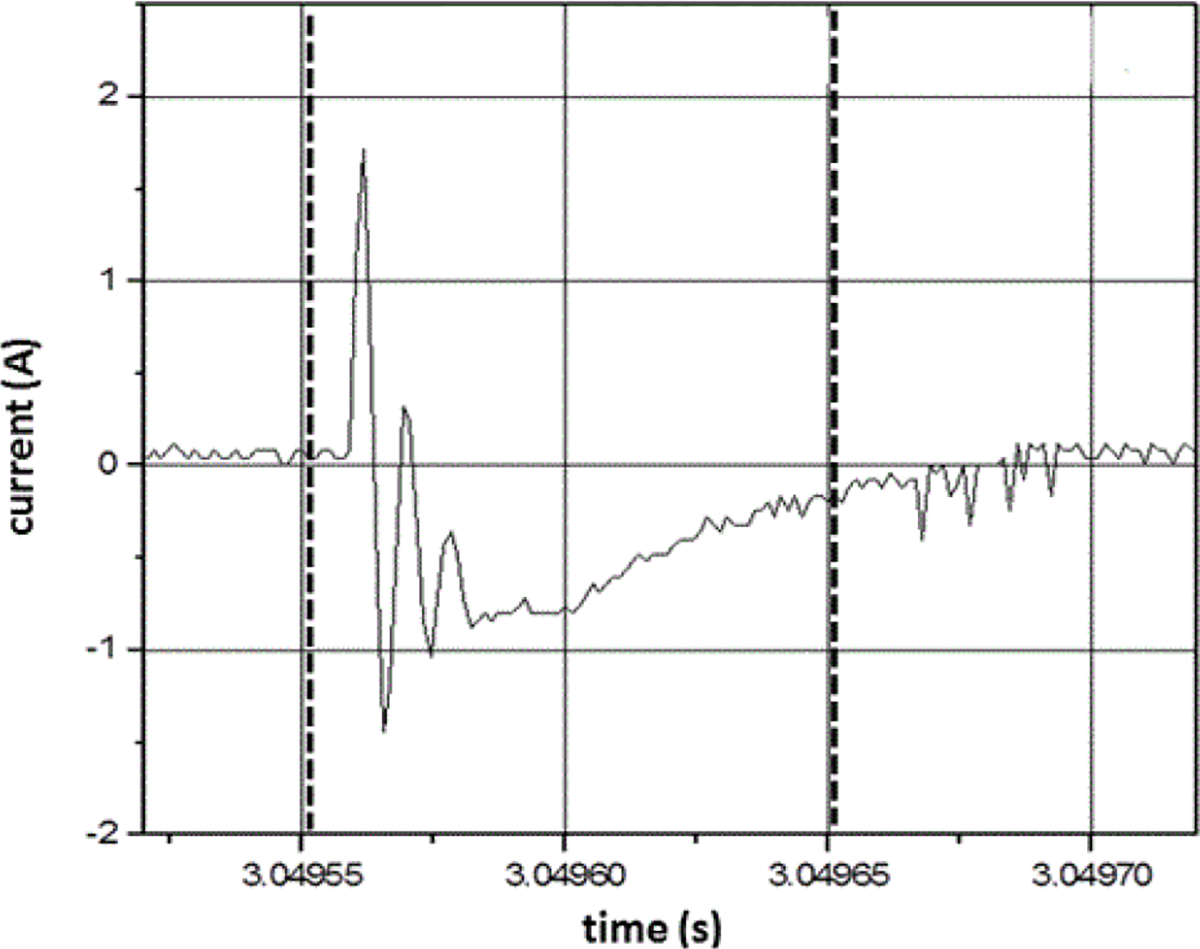
One pulse from the pulse train shown in Figure 4. The vertical dashed lines show the subset of this waveform epoch that is contained in Figure 8.

**Figure 8:**
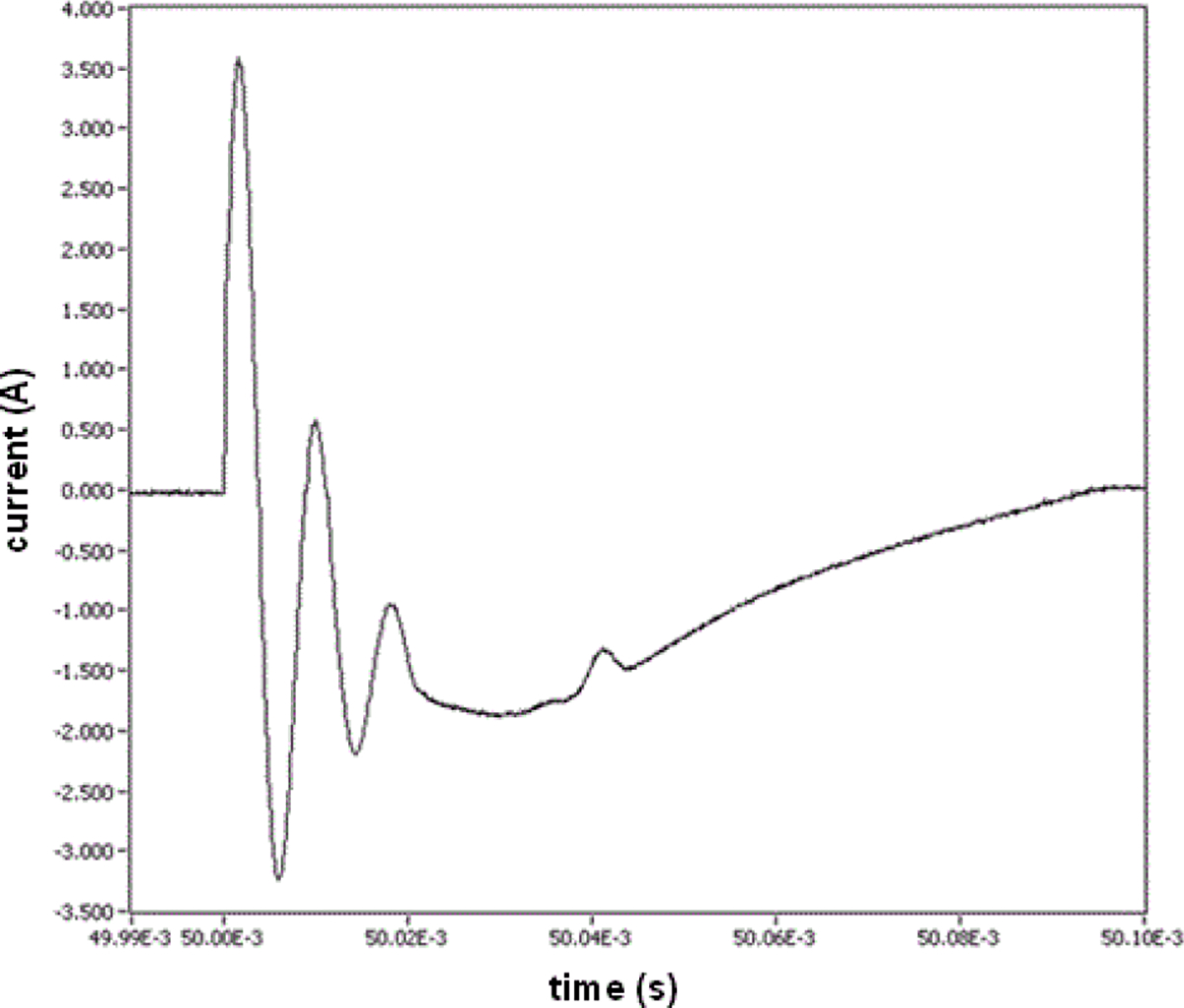
One pulse of the pulse train that was output from the same ESW that gave the waveform shown in Figure 7. In this case, the sampling interval is 12.8 ns.

Sampling rate (= 1/sampling interval) is very important to the waveform fidelity. Figures 4 and 5 show waveforms acquired with 1.28 μs and 2.5 μs sampling intervals. The variation in peak current output for the ESW shown in Figure 4 is about 10% from the mean peak current. For the high-voltage output shown in Figure 5, the peak voltage for a pulse can exceed 200% of the mean high-voltage peak. Neither of these variations is actually caused by the ESW; they are due to the sampling interval being too large to accurately capture the high-frequencies components of the output of the ESW. This is seen by comparing Figure 7, where the data is taken with a sampling interval (temporal resolution) of 1.28 μs, and Figure 8, where the sampling interval is 12.8 ns. As can be seen, the noise appears much greater in Figure 7 than in Figure 8.

This apparent greater noise in Figure 7 than in Figure 8 is because all waveforms captured from the ESW are unique single-shot events, some events contain more aberrations than others, and the sampling interval for Figure 7 was not adequate to capture the smoothly varying aberrations in the signal. The peaks in the current waveform are greater in Figure 8 than they are in Figure 7 also because of the sampling interval difference. Note, that the waveform epoch in Figure 8 is approximately 100 μs and that in Figure 7 is approximately 200 μs. Therefore, the waveform span in Figure 8 that is similar to that in Figure 7 is bound by the vertical dashed lines in Figure 7. The comparison of these figures and the waveforms shown in Figure 4 demonstrate the importance of a waveform recorder having sufficient temporal resolution to acquire waveforms with detail while at the same time capturing the entire pulse train. If the waveform recorder does not provide this capability, certain characteristics of the ESW output may not be captured. Furthermore, if these ESW output characteristics have some effect on the physiological response to an ESW shock, it would not be possible to associate an observed physiological response with a specific characteristic of the ESW output and, consequently, to adjust the ESW output to achieve the desired response.

## Test Methods

The test methods used for measurement of ESW output signals are typical of those used in the measurement of any pulse generator output. Therefore, only a brief description of how a test method is implemented is given here, the majority of this section is focused on methods for system calibration and the measurement of the electrical load. ESW testing requires measuring both the current and high-voltage output, thus one current transducer and one high-voltage are required. The high-voltage transducer is typically a high-input-impedance device and is attached in parallel to the load resistor. It provides electrical isolation between the high-voltage output of the ESW and the low-voltage input of the waveform recorder and converts its high-voltage input into a proportional low-voltage output.

The output impedance of the high-voltage transducer is matched to the input impedance of the waveform recorder and is connected to the input of the waveform recorder.

The current transducer provides electrical isolation between the high-voltage output of the ESW and the low-voltage input of the waveform recorder. The current passing through the electrical load is sensed by the transducer and provides a proportional low-voltage output. The output impedance of the current transducer is matched to the input impedance of the waveform recorder and is connected to the input of the waveform recorder.

The ESW is a single-shot device, meaning that only one pulse train event occurs and can be recorded per activation of the ESW trigger. Consequently, the waveform recorder must be operated in a mode that allows triggering from the input signal, which will be the signal going to either the channel recording the current output of the ESW or the channel recording the high-voltage output of the ESW. The waveform recorder has to be set to be continuously recording inputs on these two channels and to store the high-voltage and current waveforms into memory only after the trigger has been provided. This trigger occurs at the instant when the input signal exceeds a user-defined threshold.

Once a waveform has been acquired, it must be processed to remove the effects of the measurement system, thus providing a processed waveform that more closely approximates the actual output of the ESW than does the measured waveform. This correction process is typically done through a process called waveform reconstruction (see [9] as an example). The information necessary to perform the waveform reconstruction is obtained through calibration of the measurement system.

### System calibration

The measurement system must be calibrated to ensure accurate and reproducible acquisition of the current and high-voltage outputs of the ESW. Since both time-domain and frequency-domain parameters are used in the description of the ESW output, the ESW measurement system should be calibrated in both domains. Each domain has its benefits: the frequency domain calibration is better suited for accurate calibration of the frequency response and characteristics than is time domain calibration whereas time domain calibration is better suited for accurate calibration of temporal parameter measurements than are frequency domain techniques. Although the frequency/time transforms show the equivalence of the two domains, noise and jitter will control which domain is better suited for the particular measurement application. Lastly, calibrating the entire measurement system at once is more accurate and introduces less measurement uncertainty than calibrating each component separately and then combining the results.

**Figure 9:**
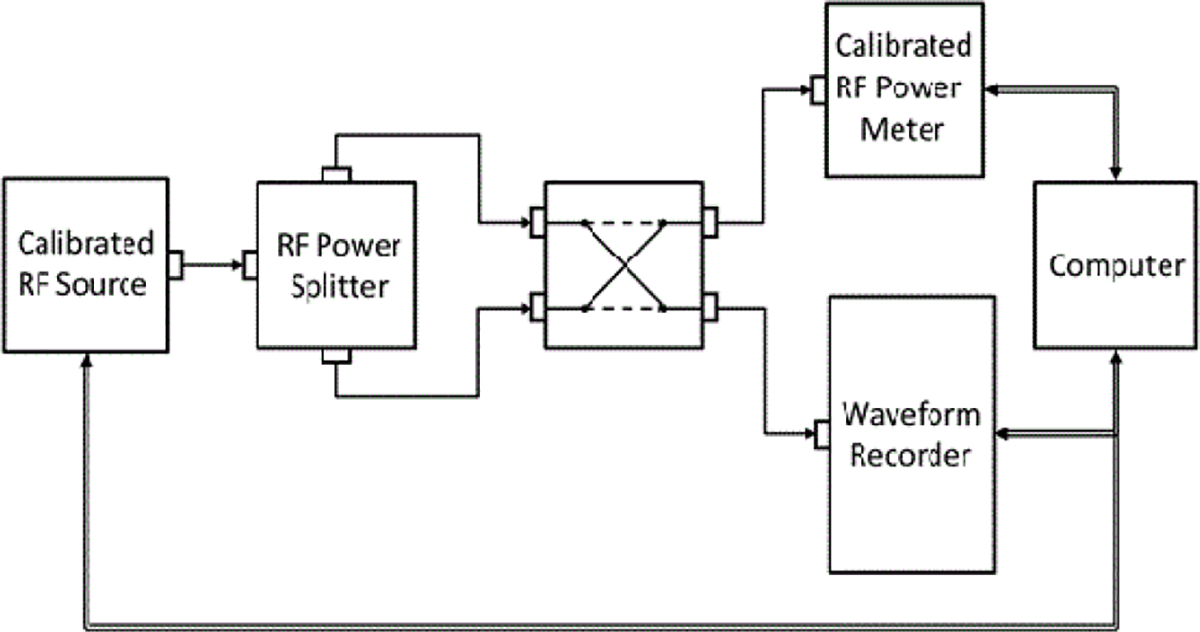
Frequency-domain ESW calibration set up.

**Figure 10:**
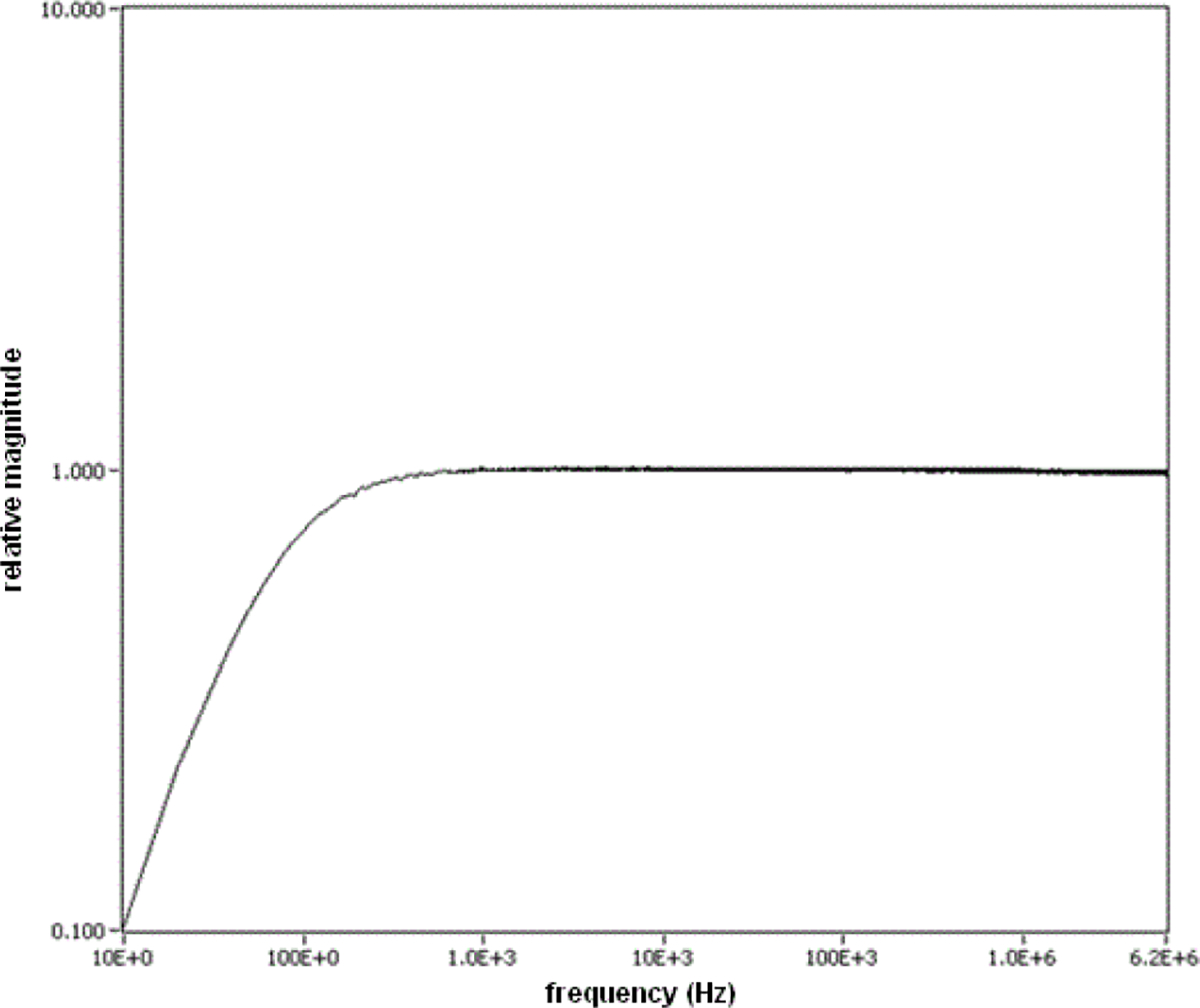
Frequency response of the current channel of an ESW measurement system, including waveform recorder, current probe, connectors, and cabling.

An example of frequency-domain method for calibrating the frequency response of the ESW measurement system is shown in Figure 9. This is a typical calibration method in which the response of the measurement system (shown in Figure 9 simply as the waveform recorder) to a stimulus is compared to the response of a calibrated power meter to the same stimulus. Swept frequency methods currently do not provide a direct measurement of the phase response because of the lack a suitable phase reference, which is a major disadvantage of the frequency-domain method. A representative frequency response of an ESW, using the system shown in Figure 9, is shown in Figure 10.

A time-domain method can be used to calibrate the ESW measurement system. In this case, a reference pulse generator would be used. The time-domain methods, through a time-frequency transform, can provide both the impulse response and the magnitude and phase responses of the ESW measurement system.

### Timebase calibration

**Figure 11:**
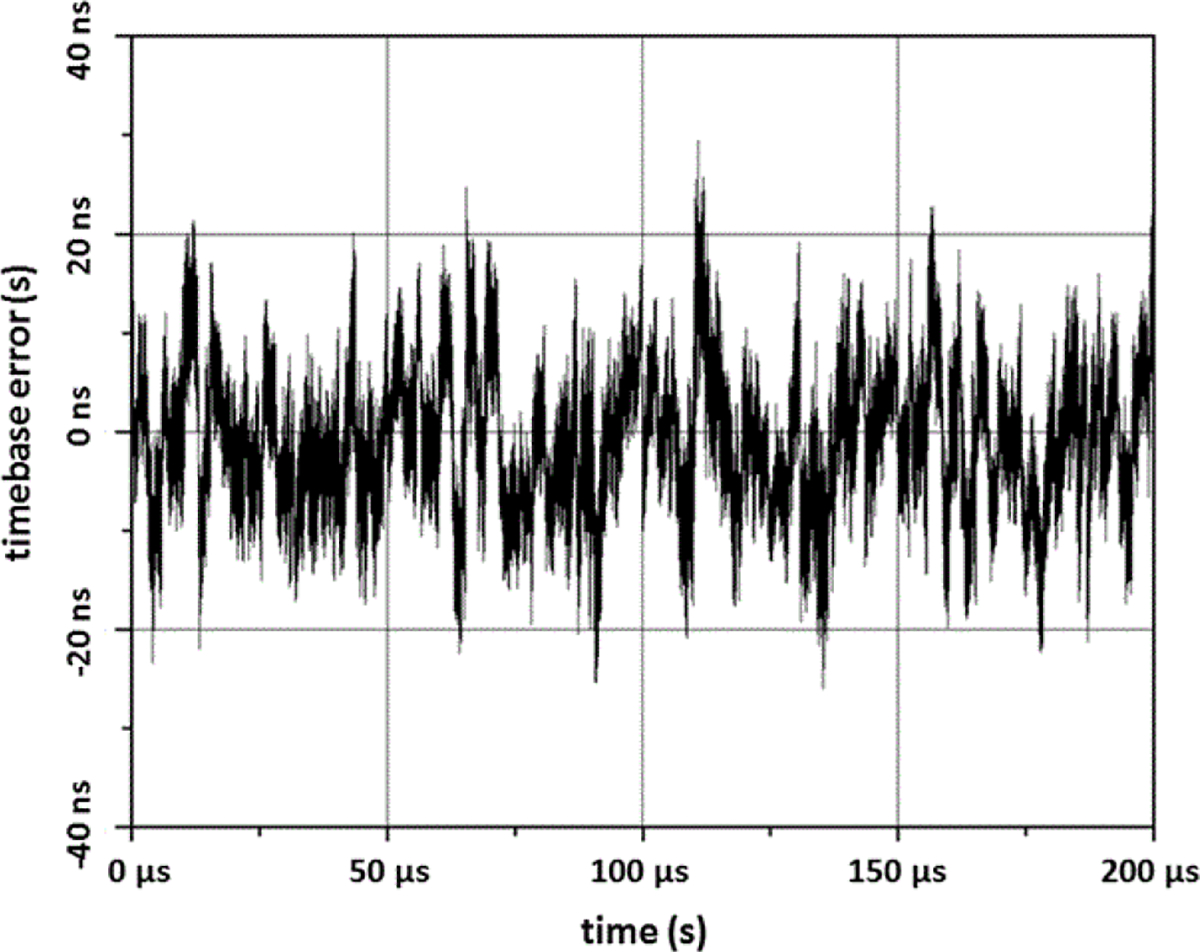
Timebase error for the waveform recorder.

Regardless of the method used to calibrate the amplitude response of the ESW measurement system, the waveform recorder may exhibit timebase errors, which are deviations of the actual sampling instants from the expected instants. For example, if a 200 μs epoch having 106 samples is acquired, it is expected that the sampling interval is uniform and equals 0.2 ns. However, this is not usually the case, as demonstrated by the waveform in Figure 11. These timebase errors will manifest themselves in both the time and frequency domain ESW performance parameters. The magnitude of the effect is dependent on the severity of the timebase errors and on the dependence of the performance parameter on the sampling intervals.

### Electrical loads

**Figure 12:**
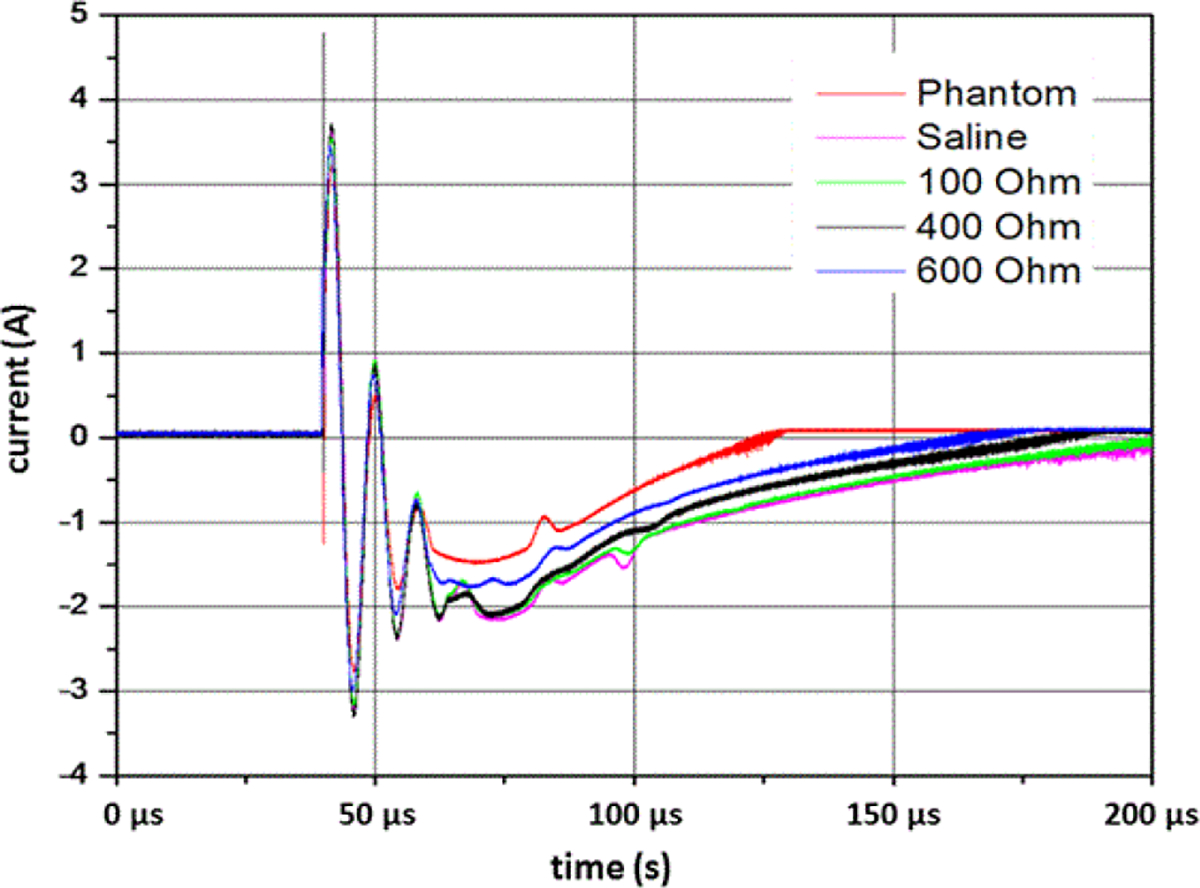
Current output of a single pulse from a pulse train into the loads indicated in the key. The electrical conductivity of the phantom and saline solution were nominaly 0.8 S/m. These waveforms were generated by the same ESW whose output current waveform is given in Figure 7. The samplling interval is 12.8 ns.

Testing of the ESW requires electrical loads that emulate the electrical load presented by the expected target. Figure 12 shows the output of a given ESW model as a function of the electrical load. The “phantom” in the figure refers to a solid polymeric (carbon-black loaded fluoropolymer) material [10] that has the same nominal bulk conductivity of human muscle tissue, which is about 0.8 S/m from about 10 Hz to 100 MHz [11,12]. The saline solution used also has an electrical conductivity of about 0.8 S/m. Both the phantom’s and saline’s electrical conductivity can be adjusted to emulate that of the human body. The reason the phantom did not perform more like the saline solution, as shown in Figure 12, is due to the contact resistance to the phantom being greater than to the saline solution. The phantom is not recommended for this application because of this contact problem. The saline solution is not recommended because of the maintenance required to maintain its required electrical conductivity. High-precision (1%) high-voltage resistors can also be used as electrical loads, as show in Figure 12. As can be observed in Figure 12, the effect of the load affects the magnitude of the current waveform and thus the calculation of the power delivered to the load (or target). Since the volume of living tissue in which the ESW current flows may include various tissues depending on the separation between ESW electrodes, the conductivity of that electrical path may vary over a wide range. Consequently, it is recommended in the IEC 62792 that three resistance values be used: 300 Ω, 600 Ω, and 1000 Ω.

### Adaptation to in operando changing electrical load

**Figure 13:**
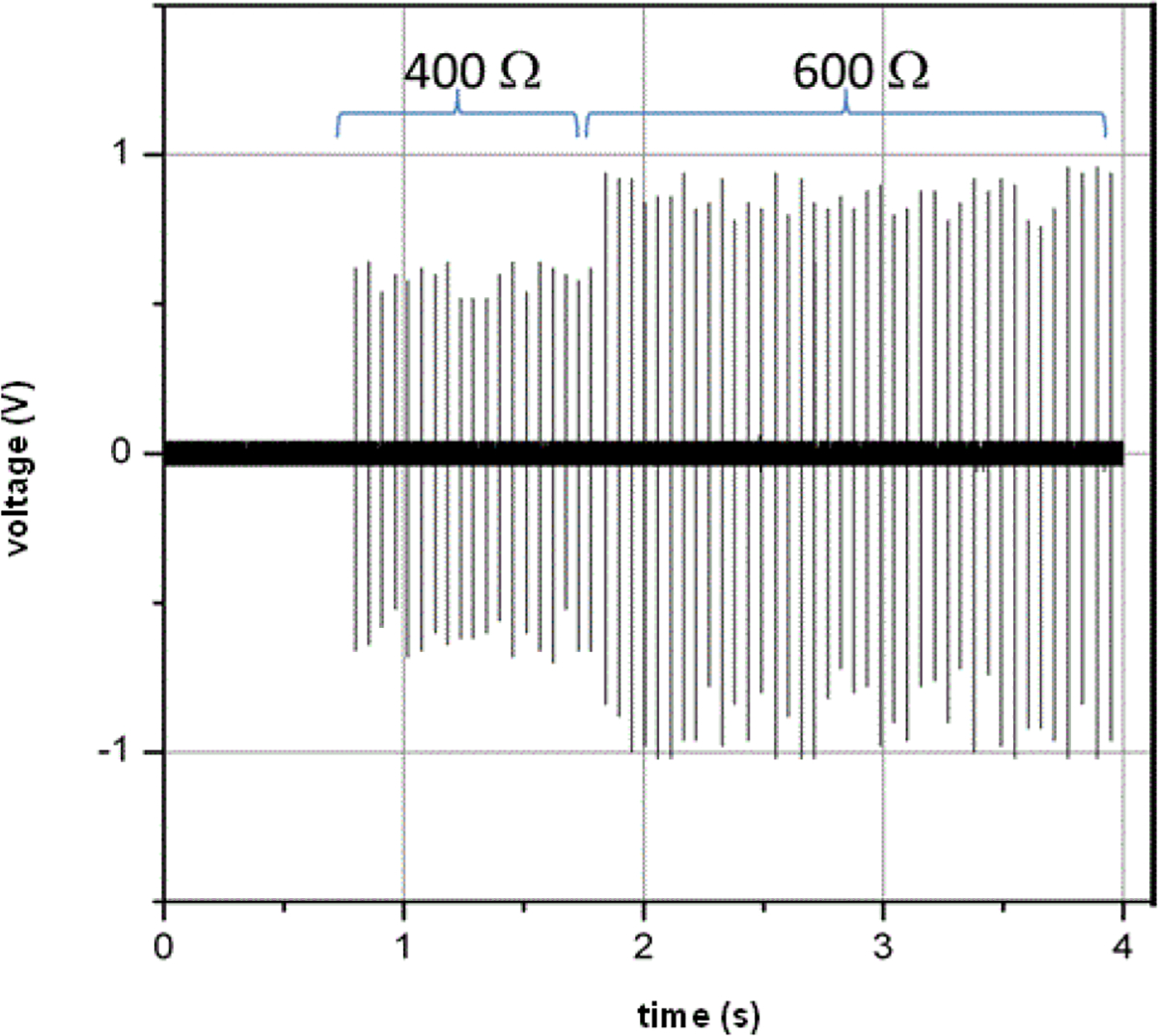
Voltage output of an ESW with an abrupt change in load between the 19th and 20th pulses in the pulse train.

Some ESW manufacturers claim that their ESW can sense changes in the electrical load presented by the target during a pulse train event and, using that information, adapt the output of the pulses in a pulse train to deliver a constant energy to the target. Therefore, measurement methods should include the capability to measure the ESW current and high-voltage output as a function of time-varying load resistances. Figure 13 demonstrates the output current of an ESW while abruptly changing the load from 400 Ω to 600 Ω between the 19th and 20th pulses.

### Metrological traceability

Metrological traceability is defined [13] as a “property of a measurement result whereby the result can be related to a reference through a documented unbroken chain of calibrations, each contributing to the measurement uncertainty of a measurement.” If the calibration of an ESW measurement system by the use of a calibration artifact (a device with accurately known characteristics) has not been performed, it will be difficult to make claims regarding the traceability of the results of the ESW measurements. Moreover, the use of an uncalibrated ESW measurement system may generate different results as compared to the results from a calibrated ESW measurement system and, without traceability, discrepancies between measurements may be difficult to resolve. Thus, metrological traceability is important to have confidence in the performance of the ESW measurement system and to help resolve measurement discrepancies.

Table 1 shows the performance specifications of three different measurement systems in the IEC 62792 [1]. The performance specifications of the reference system make such a system suitable for metrology laboratories. The secondary system shows relaxed performance specifications that are suitable for a secondary measurement system, such as which may be found in a research and development lab. The tertiary system shows the performance specifications for a system that may be used in field verification of performance. The user of the secondary and tertiary measurement systems must demonstrate metrological traceability to the reference system using artifact (transfer) standards. The calibration of each instrument in a measurement system is performed by accredited testing laboratory.

## Effects on Measurement Results

The effects of the performance characteristics of the measurement system on the ESW electrical performance parameters will be discussed in Effects of instrument performance on measurement results of this document. The effect of the values of user-defined fundamental waveform parameters, such as percent reference levels and waveform instants, on the ESW electrical performance parameters will be discussed in Effect of user-defined waveform parameters on measurement results of this document. The results presented in this section are all based on simulations to ensure knowledge of the reference values. Because these are simulation results, measurement units (V, A, s, etc.) are not necessary.

### Effects of instrument performance on measurement results

**Figure 14:**
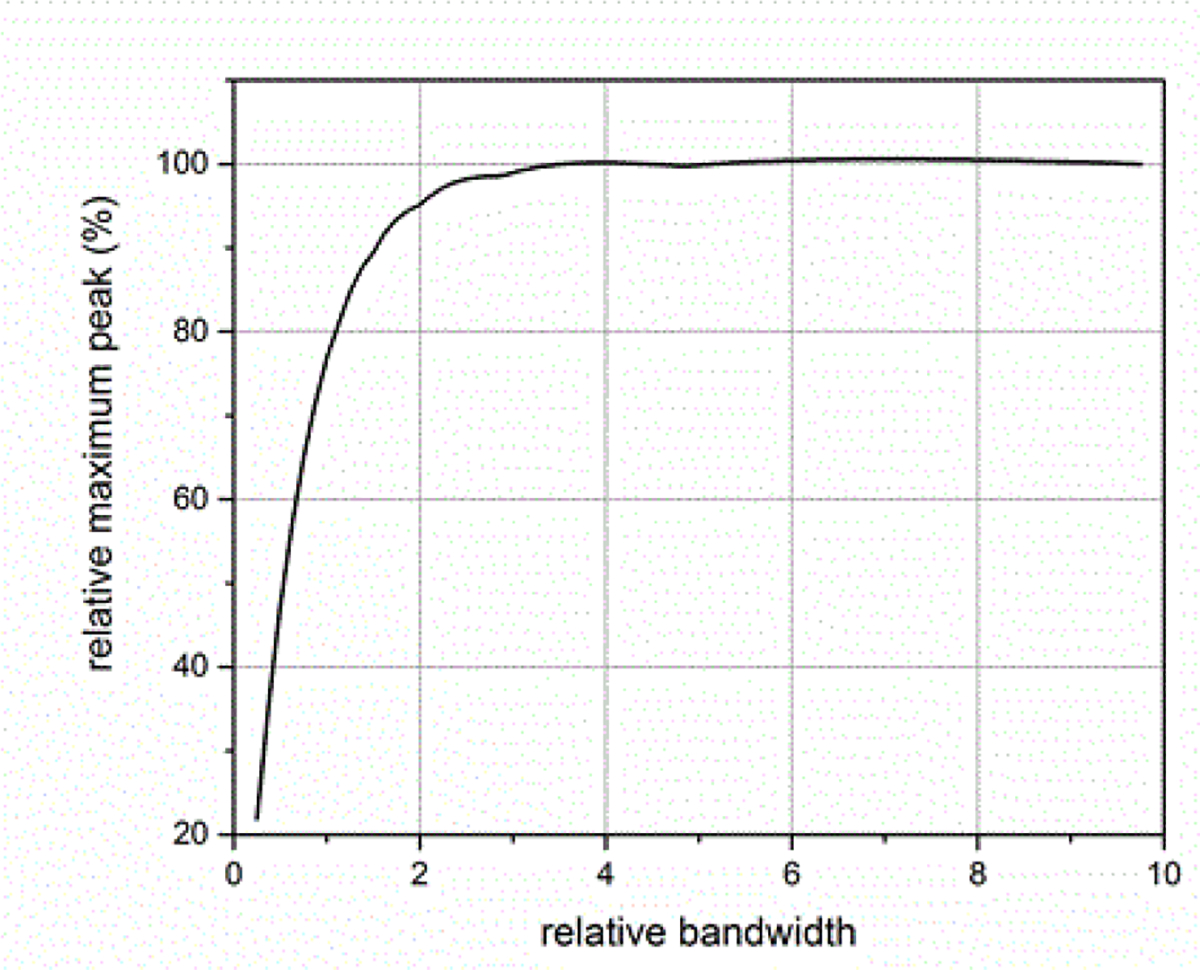
Effect of BWR on the maximum peak measured in the waveform.

**Figure 15:**
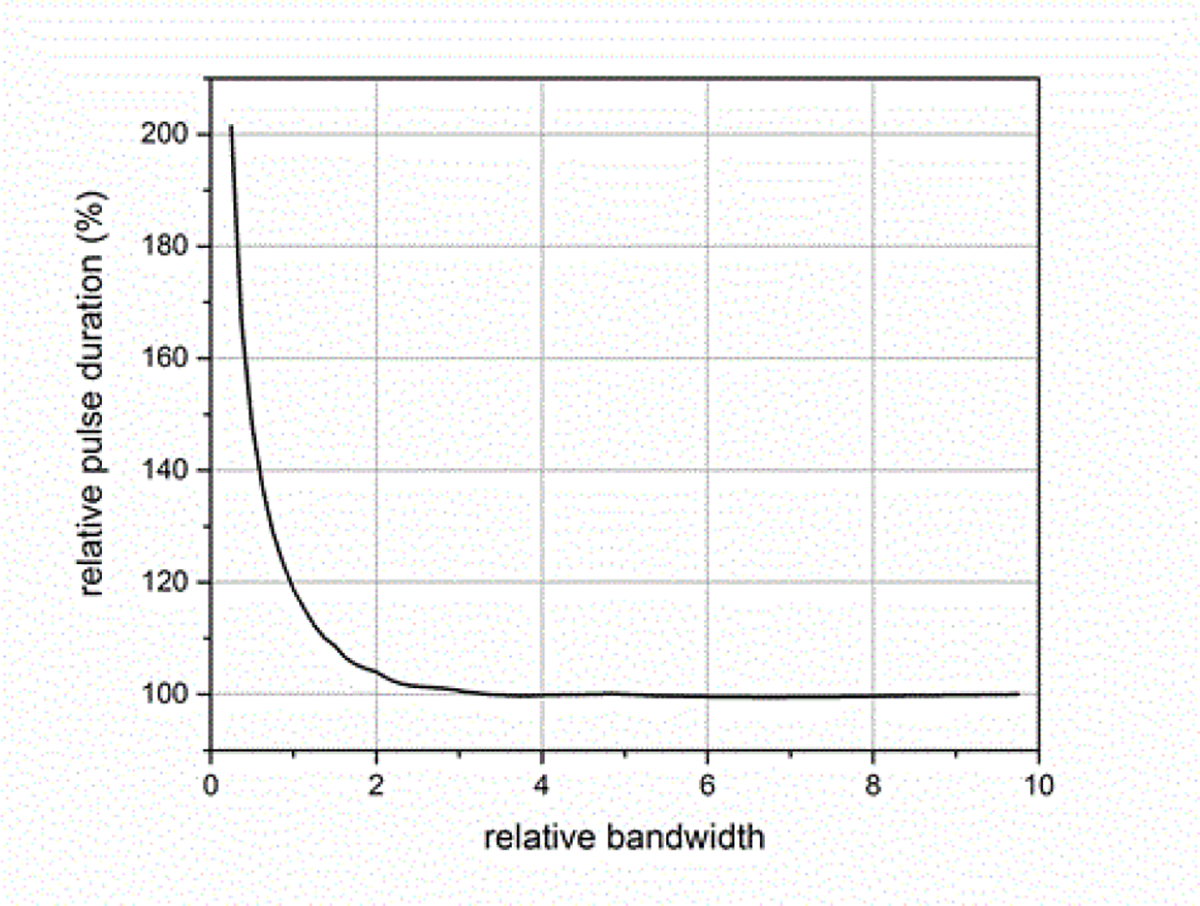
Effect of BWR on pulse duration.

**Figure 16:**
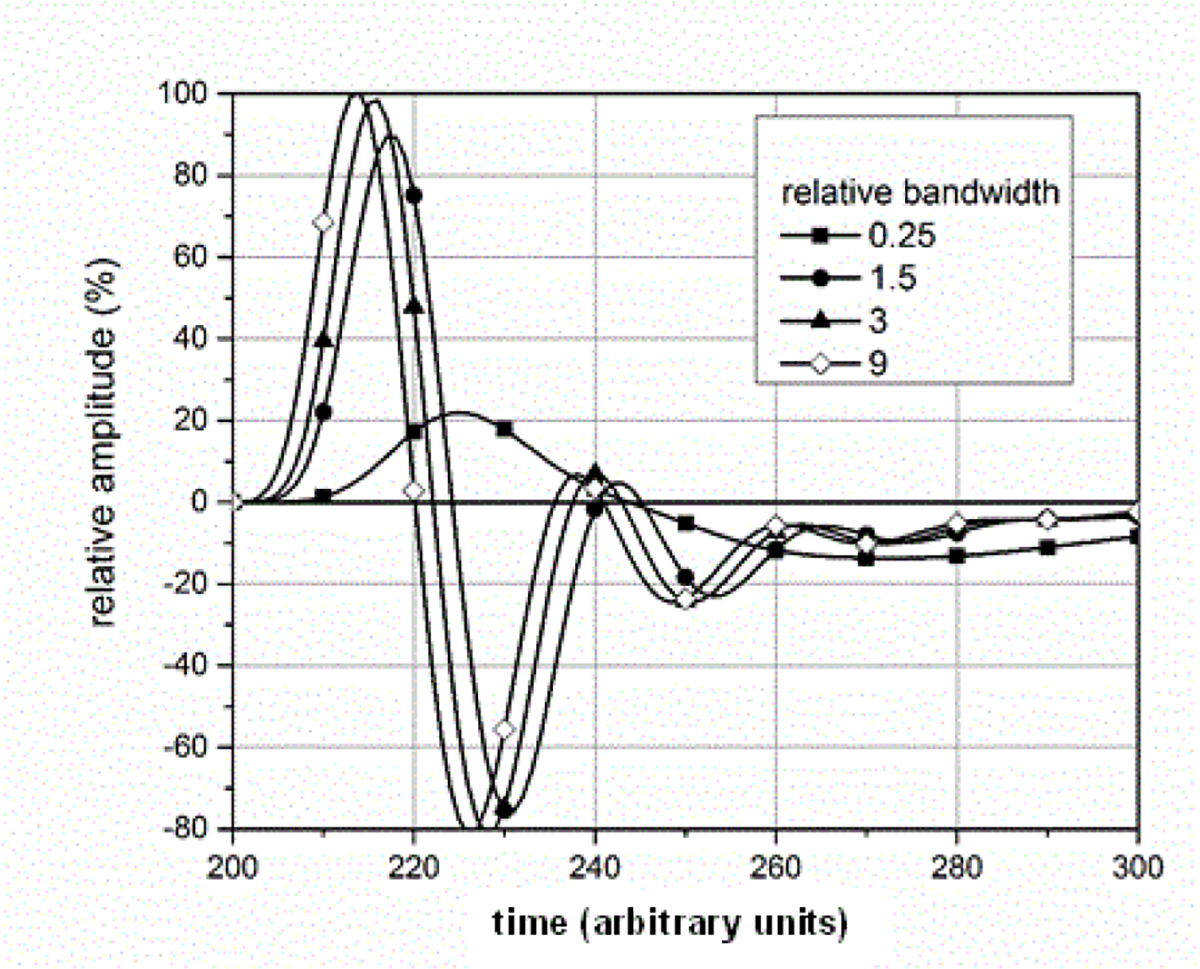
Effect of BWR on waveform shapes.

**Figure 17:**
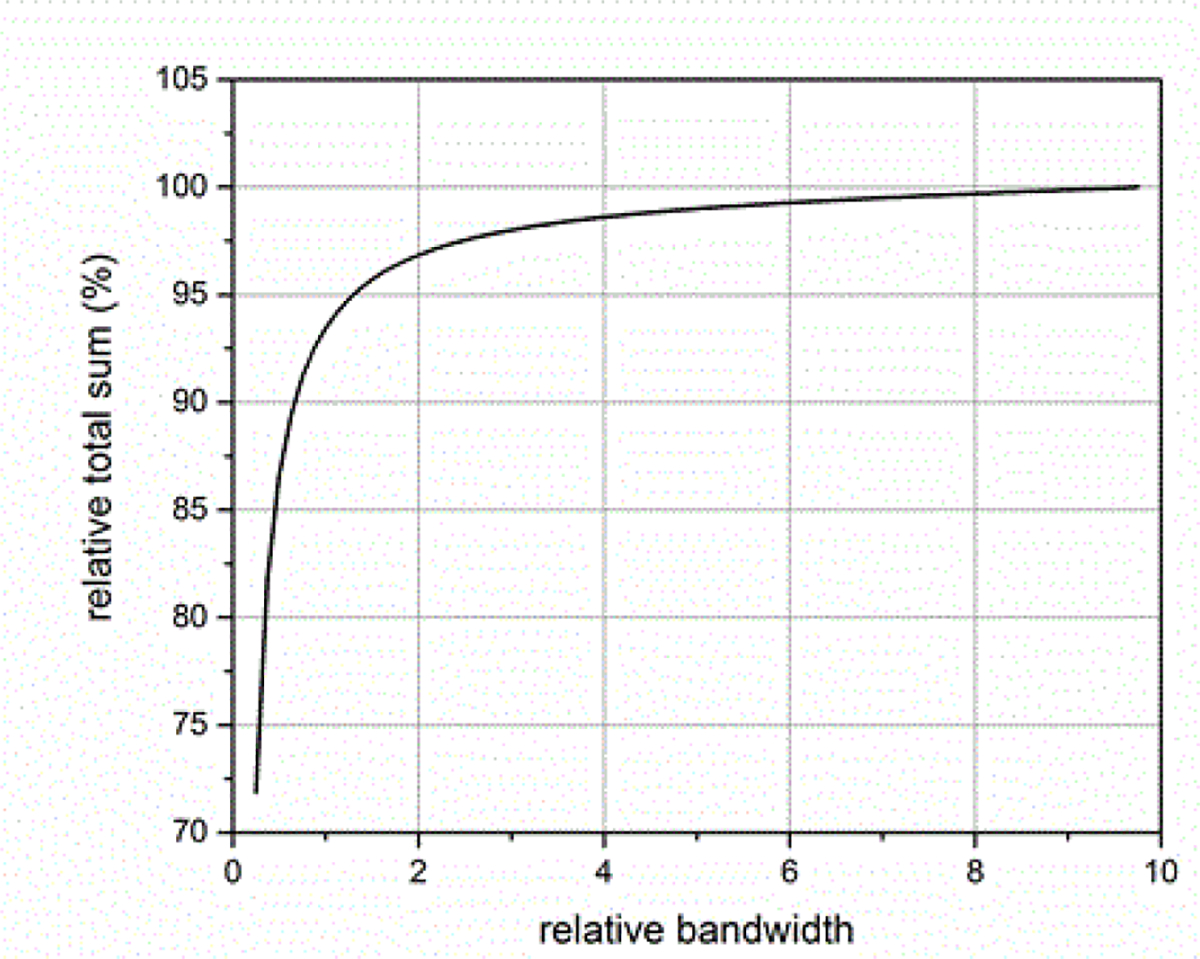
Effect of BWR on the sum of all the waveform values.

**Figure 18:**
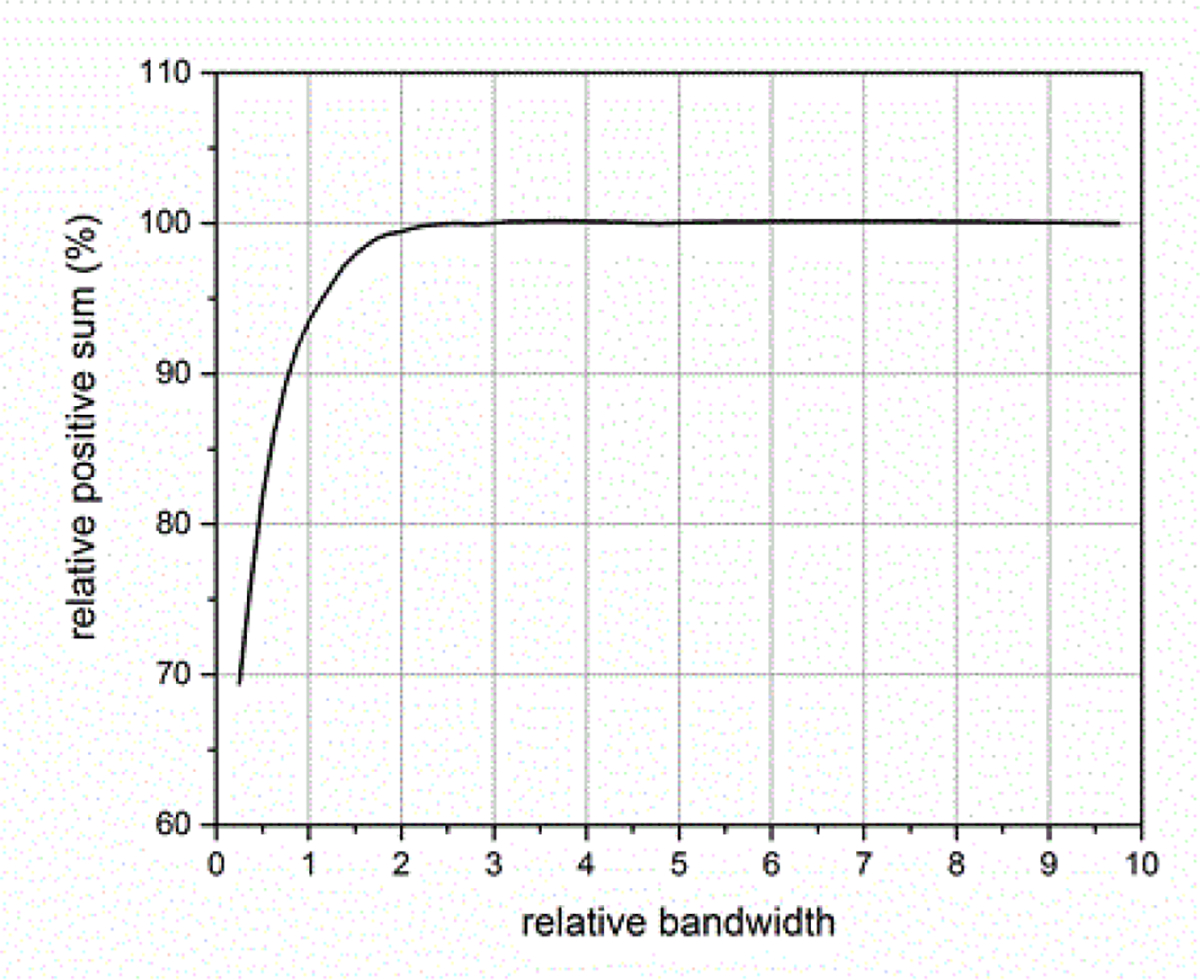
Effect of BWR on sum of positive waveform values.

The effects of the ESW measurement system’s attenuation bandwidth can affect the results obtained from the ESW measurement system. The attenuation bandwidth describes how quickly the frequency response magnitude of the measurement system decreases with increasing frequency. Typically the attenuation bandwidth is described as the frequency at which the frequency response is −3 dB (about 50 %) of the frequency response at dc (frequency = 0 Hz). Figure 14 shows the effect of the measurement system’s attenuation bandwidth on the maximum peak of the ESW output waveform. The relative bandwidth, BWR, is computed using:

(5) $$BW_{R}=\frac{BW_{\text{sig}}}{BW_{\text{sys}}}$$

where *BW*_*sig*_ is the −3 dB attenuation bandwidth of the ESW output signal and *BW*_*sys*_ is the −3 dB attenuation bandwidth of the measurement system. Figures 14–18 shown with pulse parameter values computed relative to the pulse parameter values associated with the waveform having the highest bandwidth, which are the reference values. The comparisons are presented as a percentage of the reference value. As can be seen, *BW*_*sys*_ should be greater than about four times *BW*_*sig*_ for the measurement system to have a negligible effect on the maximum peak value. Similar results apply to the minimum peak value. The bandwidth also affects the duration of a pulse, as shown in Figure 15. Figure 16 shows how the bandwidth affects the pulse shape. Bandwidth will also affect the sums (total, positive, and negative), as shown in Figures 17 and 18. The total sum corresponds to the formula in (4).

**Figure 19:**
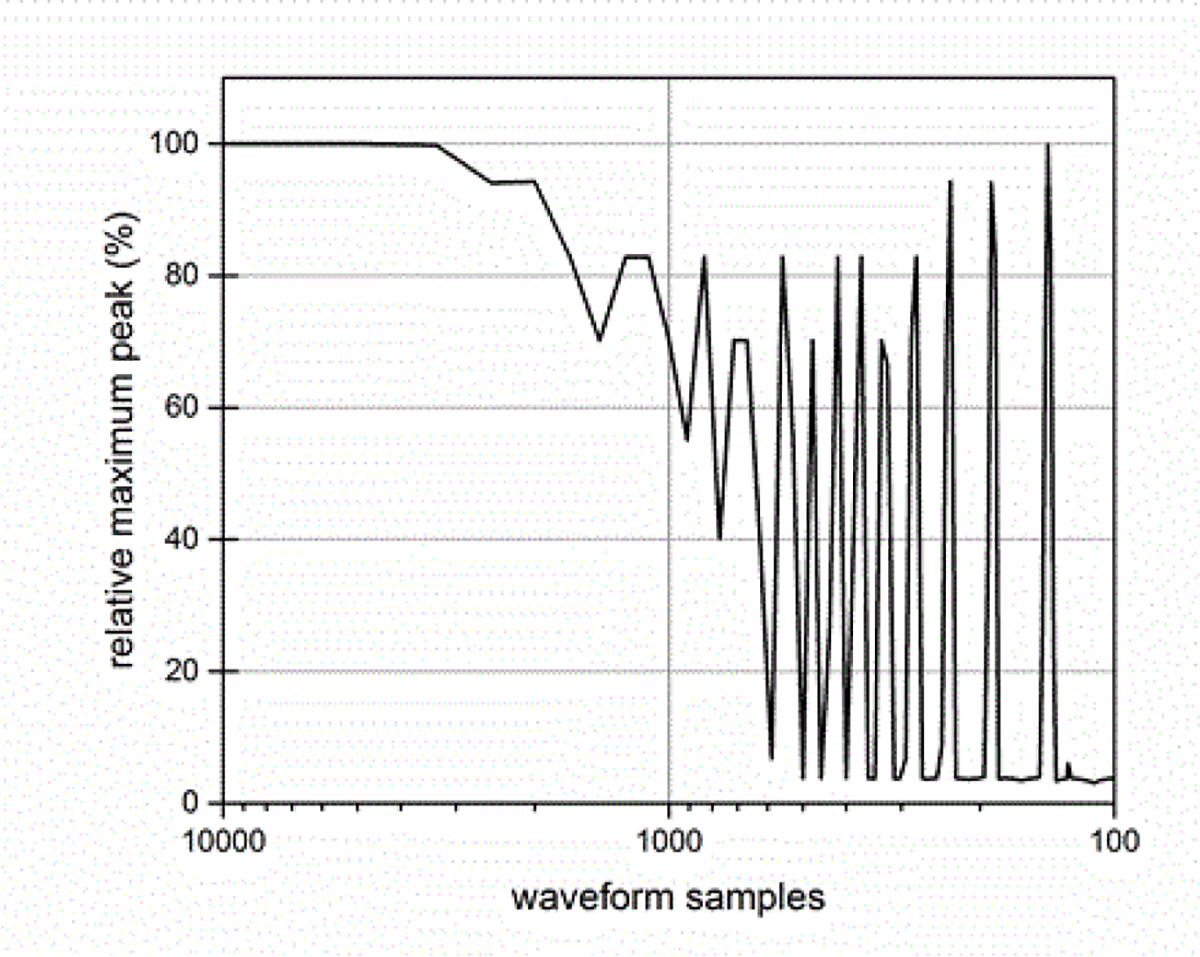
Maximum peak as a function of the number of waveform samples for a fixed duration waveform.

**Figure 20:**
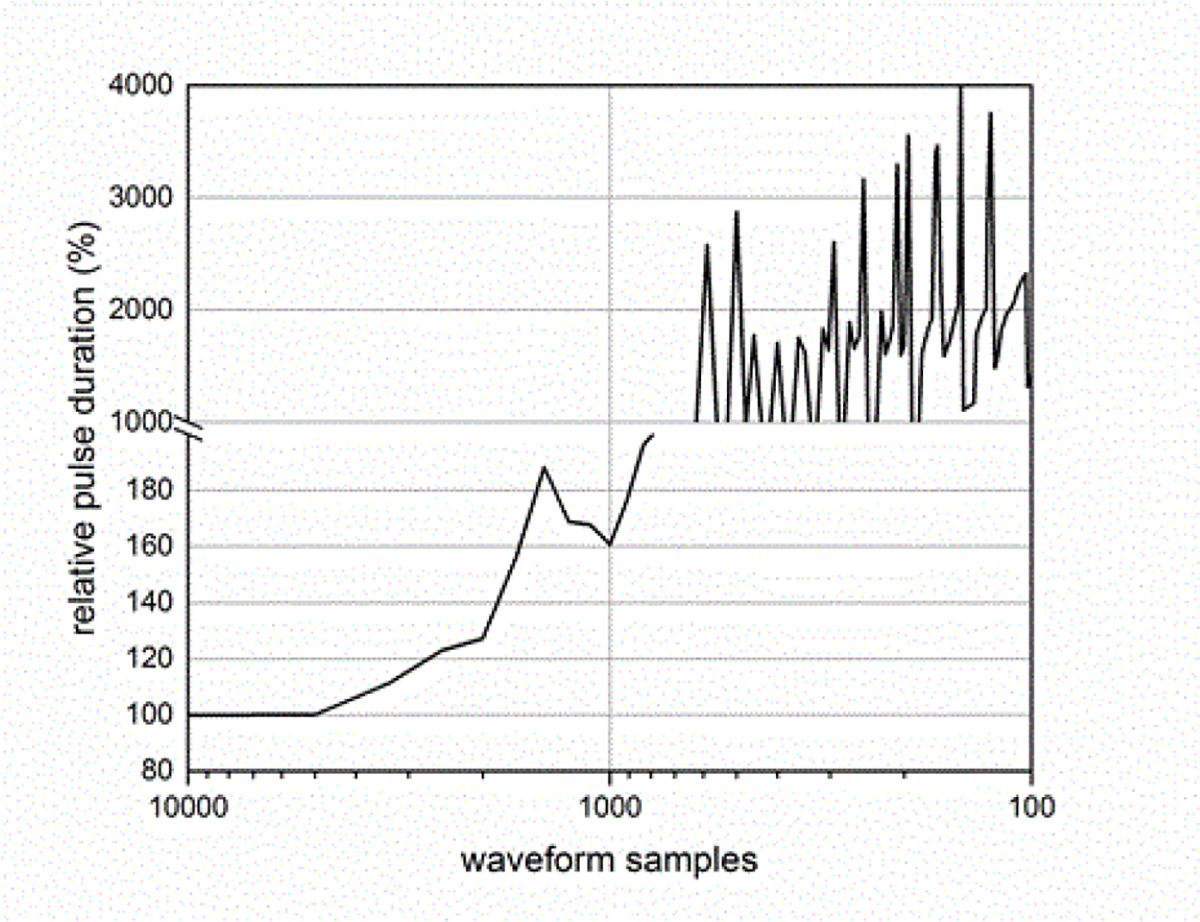
Pulse duration as a function of waveform samples for a fixed duration waveform epoch.

**Figure 21:**
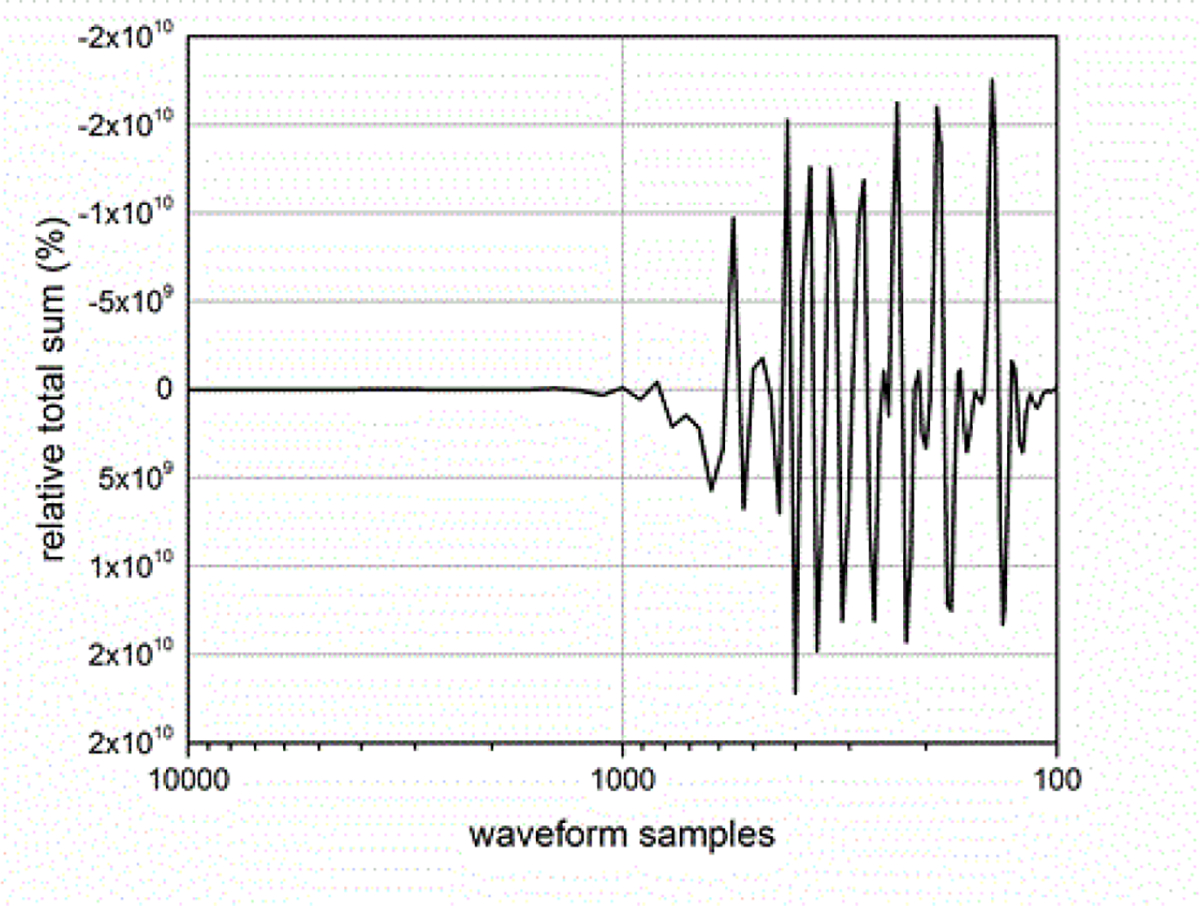
Sum of waveform samples as a function of waveform samples for a fixed duration waveform epoch.

**Figure 22:**
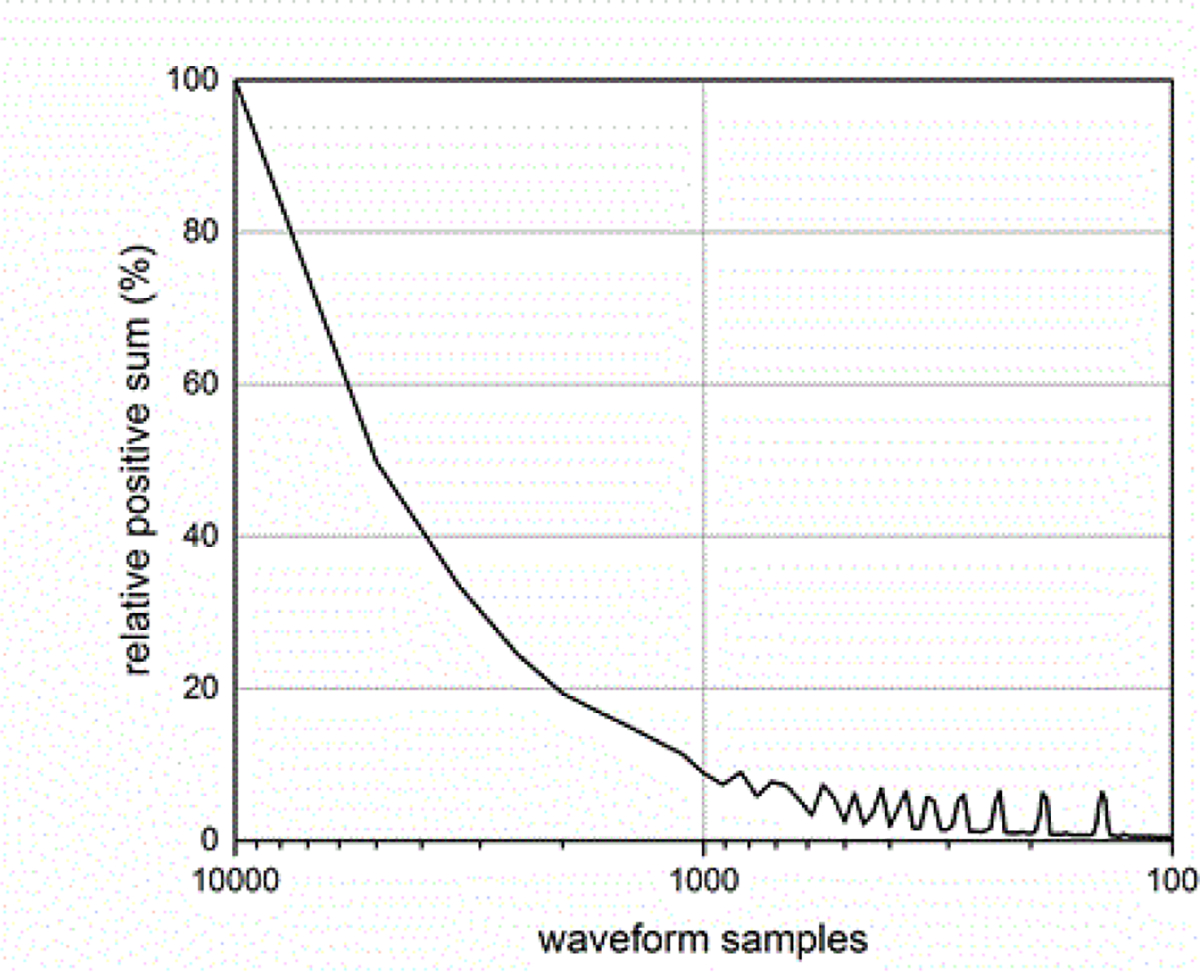
Sum of positive waveform values as a function of waveform sampling for a fixed duration waveform epoch.

**Figure 23:**
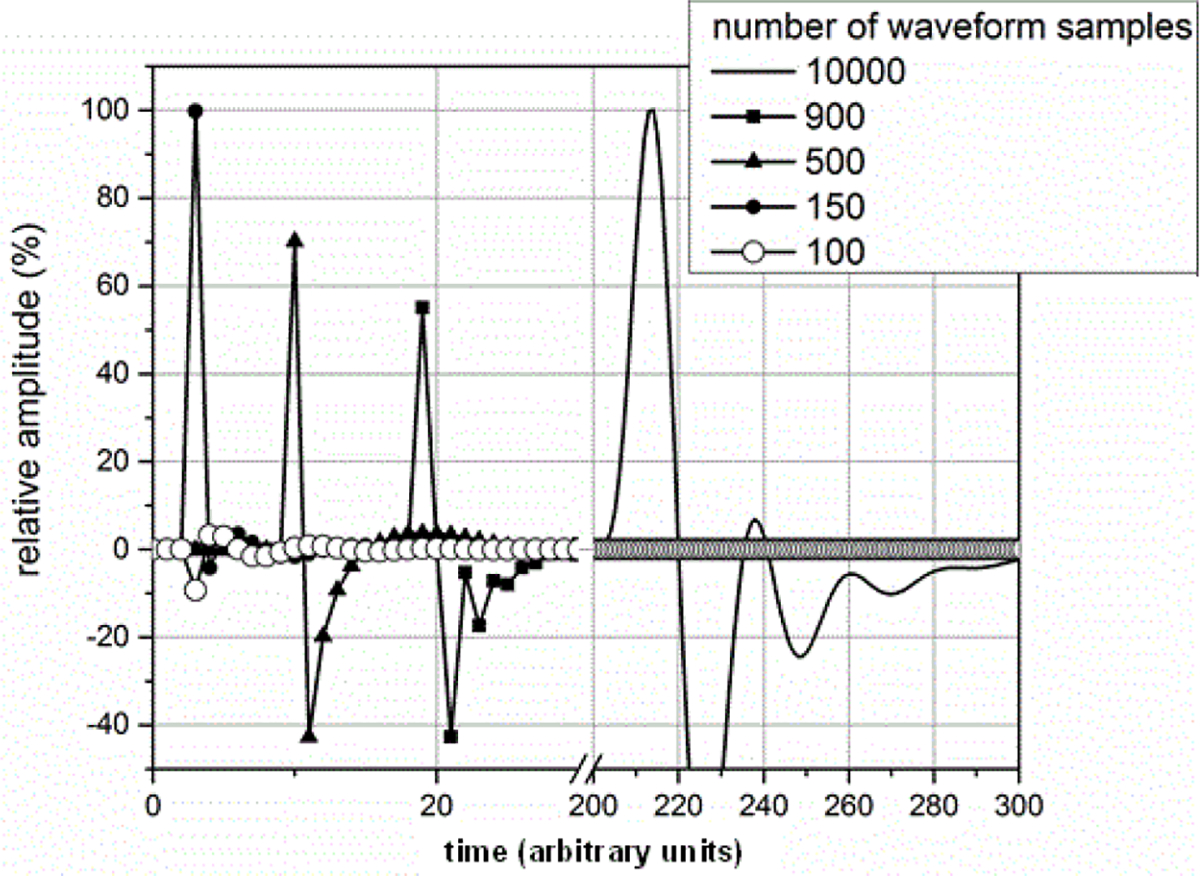
Waveform shape as a function of sampling rate. To facilitate viewing, the delays were not adjusted to reflect the different sampling intervals of each waveform.

**Figure 24:**
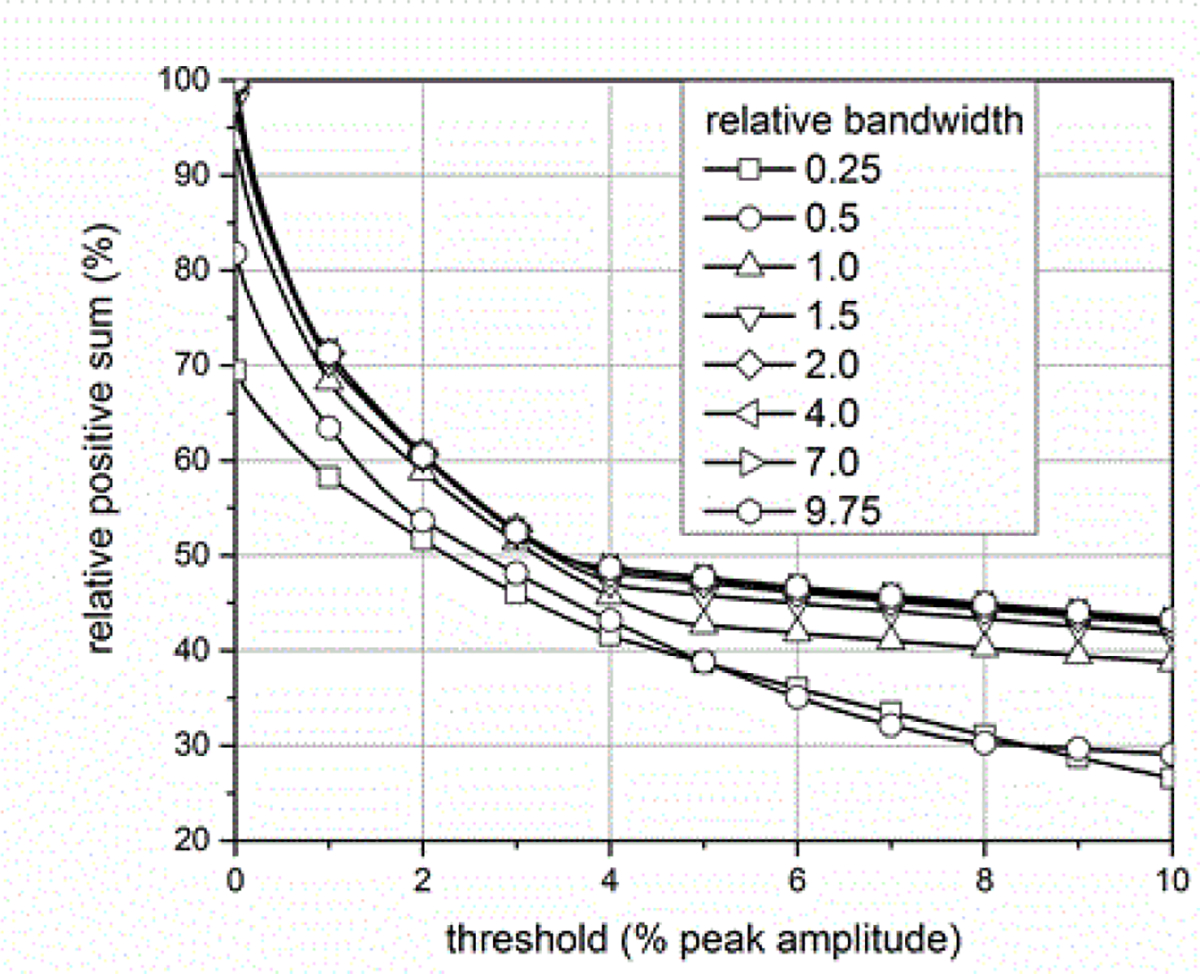
The effect of increasing the threshold for summation on the positive sum for waveforms with different relative bandwidths.

The sampling rate of the ESW measurement system can affect the results obtained from the ESW measurement system. The sampling rate describes the frequency at which the ESW output signal is measured. If the sampling rate is to low, detail of the ESW output will not be captured in the waveform and, consequently, any parameters depending on accurate temporal information will be compromised. Figures 19 and 20 show the effects of sampling rate on peak amplitude and pulse duration. The relative pulse parameter values in these figures and those in Figures 21–24 are referenced to the pulse parameter values for waveforms with 10,000 samples, which provides the highest fidelity waveform acquisition and provides the reference values. The values in the figures are presented as a percentage of the reference values.

The sampling rate was varied for a given waveform epoch. Filtering (smoothing) was not applied to reduce the amplitude discontinuity between successive samples, as this would effectively reduce the bandwidth of the measurement. Figure 21 shows the effects of sampling rate on the total sum of the waveform values, and Figure 22 shows the effect of sampling rate on the sum of the positive waveform values. The effect of sampling rate on the waveform shape is shown in Figure 23.

Noise will affect peak amplitude values as well as pulse durations. The effect of noise on pulse duration will be through the reference level instants whose values are dependent on the reference levels. The noise will affect these reference levels in two ways. First, the reference levels are based on average values and noise that is not zero mean over the user-defined epoch will affect that average value. Second, is its effect on the waveform values used in the computation of the reference level instant, *t*_*x%*_, which is computed using linear interpolation [4,5]:

(6) $$t_{x\text{\%}}=t_{x\text{\%}-}+\left(\frac{t_{x\text{\%}+}-t_{x\text{\%}-}}{y_{x\text{\%}+}-y_{x\text{\%}-}}\right)\left(y_{x\text{\%}}-y_{x\text{\%}-}\right)$$

where *t*_*x%*_ is the reference level instant for the user-selected reference level, *y*_*x%*_ is the user-selected reference level, *t*_*x%*−_ and *t*_*x%*+_ are two consecutive sampling instants corresponding to data nearest in value to *y*_*x%*_ such that *y*_*x%*−_ ≤ *y*_*x%*_≤ *y*_*x%*+,_ *y*_*x%*−_ and *y*_*x%*+_ are the two consecutive waveform values corresponding to *t*_*x%*−_ and *t*_*x%*+_. The waveform values *y*_*x%*−_ and *y*_*x%*+_ will contain noise that will directly affect *t*_*x%*_ through their effect on the denominator of equation 6 and also indirectly through their effect on the selection of their corresponding instants, *t*_*x%*−_ and *t*_*x%*+_. The effect of noise on the computation of charge will depend on how closely the sum of the noise approaches zero over the user-defined epoch.

### Effect of user-defined waveform parameters on measurement results

The user of the ESW measurement system has the option to define percent reference levels and reference instants (as described in the IEC 62792) that are used in the computation of waveform parameters, and this will affect the value of those waveform parameters. Therefore, it is important that the computation of these parameters, including the selection of reference levels and reference instants, is documented. As an example, Figure 24 shows that increasing the amplitude threshold for the summation decreases the positive sum of the waveform values. The threshold is given as percentage of the peak amplitude. The change in positive sum is the greatest for small increases in the threshold and is more pronounced for the results with higher relative bandwidth. Different waveform shapes will manifest different magnitudes of sensitivity to changes in the threshold for summation. The effects of reference instants, which would define the interval for summation, will have similar effects on this pulse parameter.

As can be seen from the data shown in Figure 24, user definitions can significantly impact the extracted waveform parameter. This, in turn, can affect the accuracy of comparisons of ESW models, of the tracking of the performance of fielded units, and of the understanding of the associated physiological effects. The determination of whether a model meets specifications can also be affected by user-defined computational or measurement variables.

Currently-available commercial-off-the-shelf oscilloscopes do not have the performance requirements to accurately capture a complete ESW waveform epoch and/or to measure waveform parameter values, which is demonstrated by Figure 14–23, even though they have been used for this purpose [14,15]. These instruments are limited in either sampling rate, analog bandwidth, or memory. Table 1 can be used to identify electronic and electrical components that can be assembled to provide a measurement system with the required performance.

## Conclusions/Next Steps

Electroshock weapons (ESWs) are used in law enforcement and military around the world to control a combative person or an assailant. ESWs function by providing a high-voltage low-current transient burst of electrical pulses that can temporarily incapacitate a target. Accurate information on the characteristics of this pulse is important for several reasons: meeting manufacturer or user performance specifications, accurate model comparison, accurate tracking history, and understanding the physiological effects of the pulse thereby promoting the advancement of the technology for its safe and effective use. The effects of the measurement system’s performance and user-defined calculation limits on ESW waveform parameters were demonstrated. These results show the sensitivity of measurement system design and user-defined computational and measurement variables on ESW waveform parameter values. Different measurement systems were described with an emphasis on high fidelity waveform acquisition, and nominal performance specifications for a high-fidelity measurement system were provided. Adherence to national and international standards can minimize the potential confusion that can be caused by the employment of arbitrary user-defined measurement systems and parameter definitions.

## Figures and Tables

**Table 1:** Waveform recorder minimum performance specifications. (from IEC 62792, Table 1).

Parameter	Reference system	Secondary system	Tertiary system
Analog bandwidth (MHz)	≥ 500	≥ 100	User defined
Sampling rate (samples/s)	≥ 1×109	≥ 2×108	User defined
Epoch, min (s)	≥ 10	≥ 2	User defined
Signal-to-noise ratio (SNR) (dB)	≥ 40	≥ 30	≥ 30
[IEEE 1057, Clause 8.3]			
Signal-to-noise-and-distortion ratio (SINAD) (dB)	≥ 40	≥ 30	≥ 30
[IEEE 1057, Clause 8.2]			
Spurious-free dynamic range (SFDR) (dB)	≥ 50	≥ 40	≥ 40
[IEEE 1057, Clause 8.8]			
Effective number of bits (ENOB) (bits)	≥ 7	≥ 6	≥ 6
[IEEE 1057, Clause 8.5]			
Input impedance: matched to probe impedance, given by *Z*_*probe*_	*Z*_*probe*_ ± 0.02 *Z*_*probe*_	*Z*_*probe*_ ± 0.05 *Z*_*probe*_,	*Z*_*probe*_ ± 0.05 *Z*_*probe*_,
Input impedance: not matched to *Z*_*probe*_	≥ 10 *Z*_*probe*_	≥ 10 *Z*_*probe*_	≥ 10 *Z*_*probe*_

**Table 2:** Connector, cable, load, and probe minimum performance specifications. (from IEC 62792).

Parameter	Reference system	Secondary system	Tertiary system	
Cable and connector impedance	50 Ω ± 2 Ω			
Cable and connector analog bandwidth	> 1 GHz			
Electrical load resistance	300 Ω ± 3 Ω 600 Ω ± 6 Ω 1000 Ω ± 10 Ω			
Electrical load inductance	<0.01 *L*_*ESW*_ or 20 nH, whichever is greater, where *L*_*ESW*_ is the self-inductance of the wire connecting the barbs and body of an ESW			
Current probe current-voltage ratio	appropriate for ESW output and input range of waveform recorder			
Current probe output impedance	*Z*_*WR*_ ± 0.05 *Z*_*WR*_ for matching to a waveform recorder with a nominal input impedance, *Z*_*WR*_ of 50Ω<0.1 *Z*_*WR*_, otherwise			
Current probe analog bandwidth	≥200 MHz	≥100 MHz		User defined
High-voltage probe hi-voltage/voltage ratio	appropriate for ESW output and input range of waveform recorder			
High-voltage probe output impedance	*Z*_*WR*_ ± 0.05 *Z*_*WR*_ for matching to a waveform recorder with a nominal input impedance, *Z*_*WR*_ of 50 Ω <0.1 *Z*_*WR*_, otherwise			
High-voltage probe analog bandwidth	≥100 MHz	≥50 MHz		User defined
